# Grubbs–Hoveyda type catalysts bearing a dicationic *N*-heterocyclic carbene for biphasic olefin metathesis reactions in ionic liquids

**DOI:** 10.3762/bjoc.11.178

**Published:** 2015-09-15

**Authors:** Maximilian Koy, Hagen J Altmann, Benjamin Autenrieth, Wolfgang Frey, Michael R Buchmeiser

**Affiliations:** 1 Lehrstuhl für Makromolekulare Stoffe und Faserchemie, Institut für Polymerchemie, Universität Stuttgart, Pfaffenwaldring 55, D-70569 Stuttgart, Germany, Fax: +49 (0)711-685-64050; 2 Institut für Organische Chemie, Universität Stuttgart, Pfaffenwaldring 55, D-70569 Stuttgart, Germany; 3 Institut für Textilchemie und Chemiefaser (ITCF) Denkendorf, Körschtalstr. 26, D-73770 Denkendorf, Germany

**Keywords:** biphasic catalysis, ionic initiators, recycling, ROMP, ruthenium

## Abstract

The novel dicationic metathesis catalyst [(RuCl_2_(H_2_ITapMe_2_)(=CH–2-(2-PrO)-C_6_H_4_))^2+^ (OTf^−^)_2_] (**Ru-2**, H_2_ITapMe_2_ = 1,3-bis(2’,6’-dimethyl-4’-trimethylammoniumphenyl)-4,5-dihydroimidazol-2-ylidene, OTf^−^ = CF_3_SO_3_^−^) based on a dicationic *N*-heterocyclic carbene (NHC) ligand was prepared. The reactivity was tested in ring opening metathesis polymerization (ROMP) under biphasic conditions using a nonpolar organic solvent (toluene) and the ionic liquid (IL) 1-butyl-2,3-dimethylimidazolium tetrafluoroborate [BDMIM^+^][BF_4_^−^]. The structure of **Ru-2** was confirmed by single crystal X-ray analysis.

## Introduction

**Figure 1:**
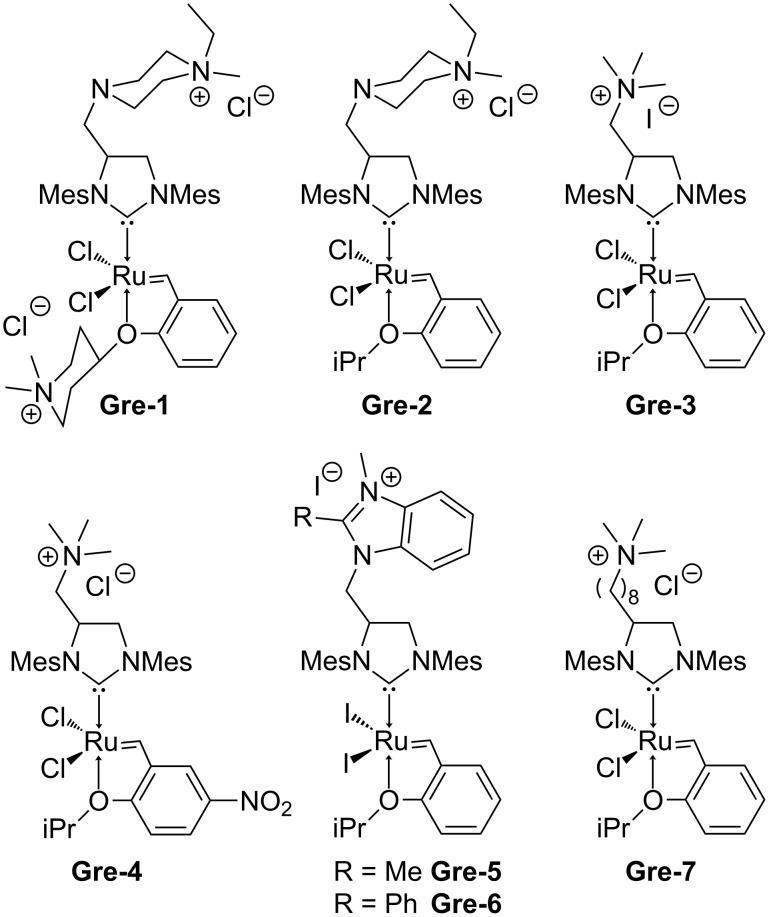
Catalysts synthesized by post-assembly tagging (Mes = mesitylene)*.*

Ionic metathesis catalysts offer access to metathesis reactions in either aqueous solution [1–10] or under biphasic conditions [11–14]. Particularly the latter aspect is of utmost relevance in case of ionic liquids (ILs) can be used as the phase in which the catalyst is dissolved. The ionic character of both the IL and the ionic catalyst effectively block any crossover of catalyst into the second (organic) phase. This offers access to metathesis reactions in which the products have a low ruthenium contamination [11]. Equally important, reactions can be run under biphasic, continuous conditions applying supported ionic liquid phase (SILP) technology [11]. We recently reported on different Ru-based ionic metathesis catalysts that can be used for these purposes. In these systems, the charge is either located directly at the ruthenium [11–12] or at the 1-methylpyridinium-4-carboxylate ligands that are introduced via anion metathesis [13–14]. These novel catalytic systems have successfully been used under SILP conditions [11,15]. Furthermore, they allow running ring-opening metathesis polymerization (ROMP) reactions under biphasic conditions, an approach that offers access to both ROMP-derived polymers with unprecedented low Ru contamination (typically 25–80 ppm) and to a regeneration of the initiator [14]. With all that systems at hand it also became apparent that reactivity of a certain catalyst strongly depends on the location of the charge. In principle, ionic Ru-based metathesis catalysts can also be prepared with the aid of *N*-heterocyclic carbenes (NHCs) that bear pendant ionic groups (Figure 1) [10,16–19]. We addressed that issue by preparing a novel ionic Ru-NHC-alkylidene using NHCs with ionic groups. Here we report our results.

## Results and Discussion

### Catalyst synthesis

**Scheme 1:**
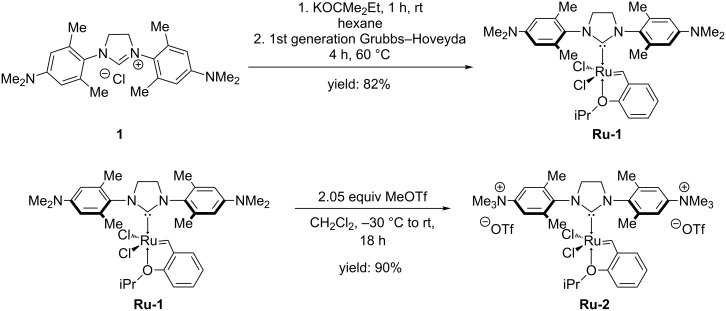
Improved synthesis of **Ru-1** and quarternization with methyl trifluoromethanesulfonate to **Ru-2**.

We were attracted by NHC ligands containing a diamino function at the aromatic ring as realized in **1** [20–22] since such ligands can be permanently quaternized to the corresponding dicationic species via double alkylation. Additionally, they remain structurally closely related to mesitylene-based NHC ligands. Attempts to synthesize ionic Ru-based olefin metathesis catalysts using imidazolinium salts bearing two quaternary ammonium groups turned out to be unsuccessful, probably due to their insolubility in common organic solvents. However, quaternization of RuCl_2_(H_2_ITap)(=CH–(2-(2-PrO-C_6_H_4_))) (**Ru-1**, Scheme 1) [21] turned out to be successful.

**Figure 2:**
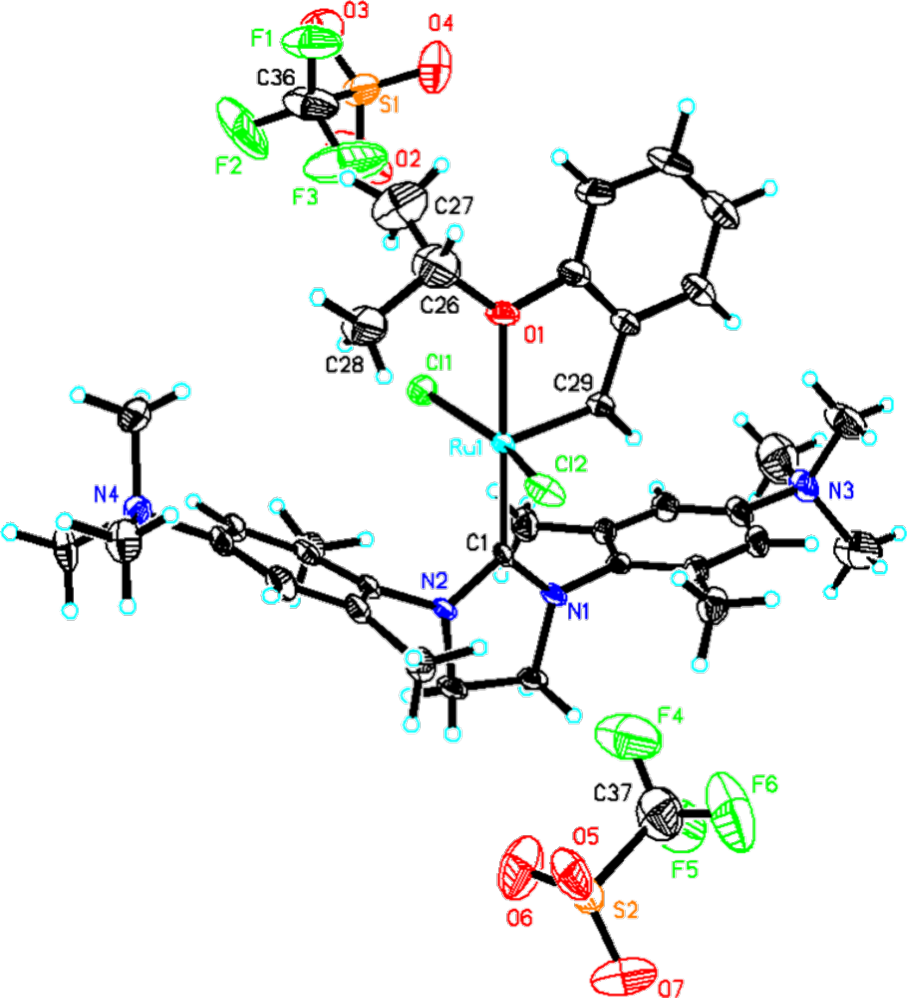
Single crystal X-ray structure of **Ru-2**. Co-solvent and disordered triflates have been omitted for clarity.

To ensure the solubility in common organic solvents until the last step of the synthesis, the neutral precursor **Ru-1** [21] was prepared in an improved one-step synthesis in 82% yield. Quarternization using 2.05 equiv of methyl trifluoromethanesulfonate gave the dicationic ruthenium alkylidene **Ru-2** in 90% isolated yield (Scheme 1). This is to the best of our knowledge the first example of a ruthenium alkylidene bearing an NHC ligand with permanent dicationic charge. Crystals of **Ru-2** suitable for single-crystal X-ray analysis were obtained from DMF/diethyl ether. Catalyst **Ru-2** crystallizes in the triclinic space group 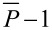, *a* = 1398.08(6) pm, *b* = 1399.41(7) pm, *c* = 1750.26(13) pm, α = 106.079(3)°, β = 112.209(3)°, γ = 99.300(2)°, *Z* = 2 (Figure 2, Supporting Information File 1).

Selected bond lengths are summarized in Table 1. For purposes of comparison, the corresponding distances of the parent system **Ru-1** are provided, too. As can be seen, the dicationic charge does influence the binding situation in **Ru-2**, though not dramatically. Interestingly only a slight increase in the Ru–NHC bond length is observed, accompanied by a very minor decrease in the Ru–O bond. The Ru–Cl bonds remain unaffected. The most dramatic effect is observed in the Ru–alkylidene bond, which is about 9 pm longer in **Ru-2** than in **Ru-1**. This increase in the alkylidene’s length points towards a substantially reduced polarization of the Ru=C bond and accounts for a reduced activity of **Ru-2** compared to standard Grubbs- and Grubbs–Hoveyda catalysts. Thus, **Ru-2** delivers only turn-over numbers well below 100 in the biphasic ring-closing metathesis (RCM) of 1,7-octadiene, diethyl diallylmalonate and *N*,*N*-diallyl *p*-toluoenesulfonamide using [BDMIM^+^][BF_4_^−^] as IL and toluene as the organic phase (see Supporting Information File 1). It is thus also in line with the fact that Ru–alkylidenes based on electron-rich NHCs, e.g., based on tetrahydropyrimidin-2-ylidenes [23], strongly promote olefin metathesis.

**Table 1:** Selected bond lengths (pm) for **Ru-2** and **Ru-1** [21].

	**Ru-2**	**Ru-1**

Ru1–C1	198.2(4)	196.6(7)
Ru1–C29	182.8(4)	173.5(9)
Ru1–O1	225.2(3)	226.0(5)
Ru1–Cl1	233.89(12)	233.0(2)
Ru1–Cl2	233.50(12)	233.9(2)

### Biphasic ring-opening metathesis polymerization (ROMP) reactions

**Figure 3:**
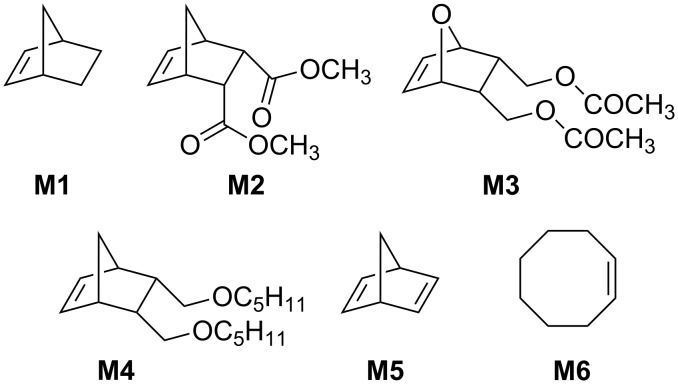
Monomers used for biphasic ROMP reactions.

To test the reactivity of **Ru-2,** various ROMP reactions were run under biphasic conditions using [BDMIM^+^][BF_4_^−^] [24] as IL and toluene as the organic phase. The structure of monomers **M1**–**M6** that were used are shown in Figure 3 [14]. Results are summarized in Table 2. At this point it is worth stressing that the purity of the IL used is of utmost importance, the more since imidazolium-based ILs can contain substantial amounts of free base [25], which in turn can negatively affect catalyst performance.

**Table 2:** ROMP reactions under biphasic conditions.^a,b^

Monomer	*T* [°C]	Time [h]	Yield [%]^c^	*M*_theo_ [g/mol]	*M*_n_ [g/mol]^d^	PDI^d^

**M1**	50	2	93	6,600	258,000	3.8
**M2**	50	2	89	14,700	94,500	2.3
**M3**	50	2	80	16,800	15,800	1.2
**M4**	50	2	86	20,600	907,000	2.8
**M5**	50	1	100	6,500	–^e^	–^e^
**M6**	70	3	36	7,700	186,000	1.50

^a^**Ru-2**, toluene, [BDMIM^+^][BF_4_^−^], 50−70 °C, 1−3 h. ^b^Ru content (measured by ICP–OES) was lower than the limit of detection, which allows for calculating a Ru content <2.5 ppm. ^c^Determined after precipitation in methanol. ^d^Measured by GPC in THF. ^e^Insoluble because of crosslinking.

As can be seen, **M1**–**M6** can be polymerized via ROMP under biphasic conditions in good yields, except for **M6**. This low yield is attributed to the comparable low ring strain in **M6**. Polydispersity indices (PDIs) were in the range of 1.2 to 3.8. Together with the high molecular weights, this is indicative for substantial chain transfer and potentially incomplete initiation of the Ru–alkylidene, particularly with unsubstituted norbornene (**M1**) and *cis-*cyclooctene (**M6**) but also with **M4**. However, in turn it allows for the synthesis of high molecular weight polymers. The most striking feature, however, is related to the Ru content of the resulting polymers, which were all obtained as white powders. Unlike in many other Ru–alkylidene-triggered metathesis-based polymerizations, Ru contamination was very low (<2.5 ppm) and even outrivals earlier reported systems bearing two pyridinium carboxylates by at least a factor of 10 [14]. Clearly, both the initiator and any Ru-containing decomposition products selectively stay in the IL phase while after termination, the polymer stays selectively in the organic (toluene) phase. Notably, polymers were simply precipitated from methanol and not subjected to any further purification steps. We believe that particularly for biomedical applications such virtually Ru-free polymers will be of utmost interest.

**Scheme 2:**
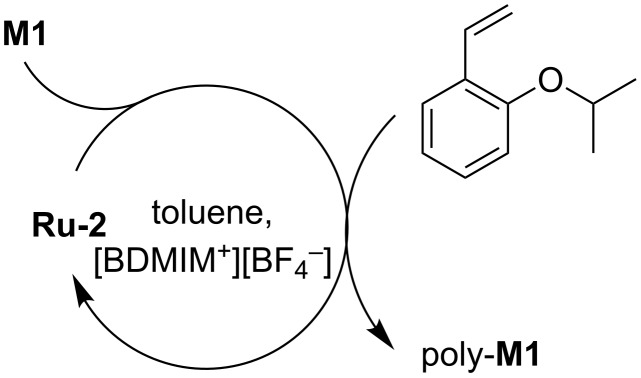
Recycling of **Ru-2** for continuous ROMP reactions.

Recycling experiments carried out with **M1** revealed that with the aid of 2-(2-PrO)-styrene, **Ru-2** could be used in three consecutive cycles (Scheme 2). Over these three cycles, the number-average molecular weight, *M*_n_, significantly decreased while PDIs increased from 2.1 to 3.0. Again, Ru leaching into the product was below the limit of detection, i.e., <2.5 ppm. The results are summarized in Table 3.

**Table 3:** ROMP of **M1** under biphasic conditions with recycling.^a^

Cycle	Yield [%]^b^	*M*_n_ [g/mol]^c^	PDI^c^

1	89	1,340,000	2.1
2	83	230,000	3.0
3	75	120,000	3.0

^a^1. **Ru-2**, **M1**, toluene, [BDMIM^+^][BF_4_^−^], 50 °C, 1.5 h; 2. 2-(2-PrO)-styrene, toluene, 50 °C, 1 h; 3. **M1**, toluene, 50 °C, 1.5 h; following cycles: repeat 2 + 3; last cycle: quenched with ethyl vinyl ether. ^b^Determined after precipitation in methanol. ^c^Measured by GPC in THF, *M*_n, theor_: 6,600 g/mol.

## Conclusion

The first dicationic Ru–alkylidene catalyst based on an *N*-heterocyclic carbene bearing two quaternary ammonium groups, [(RuCl_2_(H_2_ITapMe_2_)(=CH–2-(2-PrO)-C_6_H_4_))^2+^ (OTf^−^)_2_] (**Ru-2**), was prepared from the neutral precursor RuCl_2_(H_2_ITap)(=CH–2-(2-PrO)-C_6_H_4_)) (**Ru-1**) and methyl trifluoromethanesulfonate. Also, an improved, high-yield synthesis of **Ru-1** has been presented. **Ru-2** was tested for its reactivity in ROMP under biphasic conditions using [BDMIM^+^][BF_4_^−^] as the ionic liquid and toluene as the organic solvent. While **Ru-2** showed low RCM activity, it turned out to be active in ROMP reactions of strained cyclic olefins like norbornenes, 7-oxanorbornenes, norbornadiene and *cis*-cyclooctene allowing for the synthesis of the corresponding polymers with unprecedented low metal contamination (<2.5 ppm) without any additional purification steps.

## Experimental

**General:** Unless noted otherwise, all manipulations were performed in a Labmaster 130 glovebox (MBraun; Garching, Germany) or by standard Schlenk techniques under N_2_ atmosphere. CH_2_Cl_2_ and toluene were purchased from J. T. Baker (Devender, Netherlands) and were dried by using an MBraun SPS-800 solvent purification system. Hexane was purchased from VWR and distilled from sodium/benzophenone under N_2_. Starting materials were purchased from ABCR, Aldrich, Alfa Aesar, Fluka and TCI Europe and used without further purification. KOCMe_2_Et was purchased from Alfa Aesar as a 25 wt % solution in toluene. Toluene was co-evaporated with pentane in vacuo prior to use.

NMR spectra were recorded on a Bruker Avance III 400 spectrometer in the indicated solvent at 25 °C and are listed in parts per million downfield from tetramethylsilane as an internal standard. IR spectra were measured on a Bruker ATR/FT-IR IFS 128. GPC measurements were carried out on a system, consisting of a Waters 515 HPLC pump, a Waters 2707 autosampler, Polypore columns (300 × 7.5 mm, Agilent technologies, Böblingen, Germany), a Waters 2489 UV–vis and a Waters 2414 refractive index detector. For calibration, polystyrene standards with 800 < *M*_n_ < 2,000,000 g/mol were used. ICP–OES measurements were carried out using a Spectro Acros device (Ametek GmbH; Meerbusch, Germany). Calibration was done with Ru standards containing 0.1, 0.5, 1.0 and 5.0 ppm. Mass spectra were recorded on a Bruker Daltonics Microtof Q mass spectrometer at the Institute of Organic Chemistry at the University of Stuttgart. 1,3-Bis(2,6-dimethyl-4-dimethylaminophenyl)-4,5-dihydroimidazol-2-ylidene (**1**) [21], 2-(2-PrO)-styrene [23–24], **M3** [26] and **M4** [27] were prepared according to the literature.

**RuCl_2_(H_2_ITap)(=CH-2-(2-PrO)-C_6_H_4_-O) (Ru-1)** [27–28]: Inside a glovebox, **1** (281 mg, 0.70 mmol), KOCMe_2_Et (88 mg, 0.70 mmol) and hexane (9 mL) were added to a 50 mL Schlenk flask equipped with a magnetic stir bar. The reaction mixture was allowed to stir for 1 h at room temperature, during which time a brownish orange suspension formed. The 1st-generation Grubbs–Hoveyda catalyst (400 mg, 0.67 mmol) dissolved in hexane (6 mL) was added to the reaction mixture. The reaction mixture was removed from the glovebox and was heated to 60 °C for 4 h. The formation of a green solid was observed. After cooling to room temperature, all solids were filtered off and washed with pentane (3 × 10 mL) and diethyl ether (3 × 10 mL); then the product was redissolved in CH_2_Cl_2_. Purification was accomplished by chromatography using silica G60 and CH_2_Cl_2_/hexane. Drying in vacuo gave the product as a dark green solid (375 mg, 0.55 mmol, 82%). Analytical data were in accordance with the literature [20].

**[(RuCl_2_(H_2_ITapMe_2_)(=CH–2-(2-PrO)-C_6_H_4_))^2+^ (OTf^-^)_2_] (Ru-2):** At −30 °C, methyl trifluormethanesulfonate (99 mg, 601 µmol) dissolved in CH_2_Cl_2_ (3 mL) was added to **Ru-1** (200.5 mg, 293 µmol) dissolved in CH_2_Cl_2_ (5 mL). The mixture was stirred for 18 h at room temperature and then, CH_2_Cl_2_ was removed under reduced pressure. The residue was washed with CH_2_Cl_2_ (3 × 3 mL) and ethyl acetate (3 × 3 mL), allowing for the isolation of the target compound as a light-green solid (267 mg, 264 µmol, 90%). ^1^H NMR (DMF-*d*_7_) δ 16.47 (s, 1H, Ru=CH), 8.17 (s, 4H, NHC-Ar), 7.70–7.66 (m, 1H, C_6_H_4_), 7.20 (d, *J* = 8.4 Hz, 1H, C_6_H_4_), 7.06–7.04 (m, 1H, C_6_H_4_), 6.96 (t, *J* = 7.4 Hz, 1H, C_6_H_4_), 5.10 (hept, *J* = 7.4 Hz, 1H, O-C*H*-(CH_3_)_2_), 4.42 (s, 4H, N-CH_2_), 4.00 (s, 18H, N-(CH_3_)_3_), 2.66 (s, 12H, NHC-Ar-CH_3_), 1.27 (d, *J* = 6.1 Hz, 6H, O-CH-C*H*_3_); ^13^C NMR (DMF-*d*_7_) δ 292.9 (Ru=CH), 211.6 (N-C=N), 153.5, 148.7, 145.9, 143.1, 141.2, 131.3, 123.6, 123.5, 121.8, 114.5 (C_6_H_2_, C_6_H_4_), 127.3, 124.1, 120.9, 117.7 (CF_3_-SO_3_^−^, q, *J*_C-F_ = 322.5 Hz), 76.4 (*C*H(CH_3_)_2_), 57.9 (N(CH_3_)_3_), 52.6, 21.9, 20.3 (CH(*C*H_3_)_2_, Ar-CH_3_); ^19^F NMR (DMF-*d*_7_) δ −78.5; FTIR (ATR, cm^−1^) 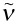: 1589 (s), 1491 (m), 1251 (m), 1152 (m), 1114 (m), 1028 (s), 923 (m), 840 (s), 801 (s), 754 (s), 637 (s), 572 (s), 517 (s) cm^-1^; MS (ESI) *m*/*z*: calcd. for C_35_H_50_Cl_2_N_4_ORu (dication, *z* = 2): 357.1199, found: 357.1214; *m*/*z* calcd. for C_36_H_50_F_3_Cl_2_N_4_O_4_RuS: 863.1923, found: 863.1905. Crystals suitable for X-ray diffraction were obtained by layering diethyl ether over a solution of **Ru-2** in anhydrous DMF.

**General ROMP-procedure: Ru-2** (5.6 mg, 5 µmol or 11.13 mg, 10 µmol) and [BDMIM^+^][BF_4_^−^] (400 mg) were placed inside a flame-dried Schlenk tube (25 mL) equipped with a magnetic stir bar. The reaction mixture was heated to the indicated temperature. The monomer (350 µmol or 700 µmol) and toluene (2 mL) were added to a separate flame-dried Schlenk tube. The monomer solution was added via syringe in one portion and the reaction mixture was allowed to stir at the indicated temperature for the indicated time. After cooling to room temperature, ethyl vinyl ether (1 mL) was added and the reaction mixture was allowed to stir for another 30 min. Finally, the reaction mixture was poured into methanol. The polymer was obtained as a white or off-white solid.

**General ROMP-procedure with recycling: Ru-2** (11.1 mg, 10 µmol) and [BDMIM^+^][BF_4_^−^] (400 mg) were placed inside a flame-dried Schlenk tube (25 mL) equipped with a magnetic stir bar. The reaction mixture was heated to the indicated temperature. **M1** (65.9 mg, 700 µmol) and toluene (5 mL) were added to a separate flame-dried Schlenk tube. The monomer solution was added via syringe in one portion and the reaction mixture was allowed to stir at 50 °C for 1.5 h. Then a solution of 2-(2-PrO)-styrene in anhydrous toluene (1 mL, 1 M) was added. The reaction mixture was stirred at 50 °C for 1 h. The two phases were allowed to separate. The organic phase was poured into methanol. The IL phase was extracted with toluene (4 × 2 mL). The extracted organic phases were also poured into methanol. Poly-**M1** was obtained as a white solid. New **M1** was added to the IL phase and the procedure was repeated. After the last cycle, the reaction was quenched with ethyl vinyl ether (1 mL).

**ICP–OES measurements:** The corresponding polymer (20 mg) was added to high-pressure Teflon tubes. Digestion was performed under microwave conditions using aqua regia (10 mL). The mixture was cooled to room temperature, diluted with deionized water (approx. 40 mL), filtered and subjected to ICP–OES for Ru with λ = 240.272 nm ion line and background lines at λ_1_ = 240.254 nm and λ_2_ = 240.295 nm.

**Poly-M1:****^1^H NMR (CDCl_3_) δ 5.35 (s, 1H), 5.21 (s, 1H), 2.79 (bs, 1H), 2.44 (bs, 1H), 1.88–1.79 (2bs, 3H), 1.35 (bs, 2H), 1.09–1.02 (m, 2H); ^13^C NMR (CDCl_3_) δ 134.1, 134.0, 133.9, 133.3, 133.2, 133.0, 43.6, 43.3, 42.9, 42.2, 41.5, 38.8, 38.6, 33.3, 33.1, 32.5, 32.4; FTIR (ATR, cm^−1^) 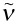: 2941 (s), 2863 (m), 1446 (w), 1260 (s), 1189 (w), 1081 (s), 1020 (s), 965 (s), 862 (w), 798 (s), 737 (m), 688 (w).

**Poly-M2:****^1^H NMR (CDCl_3_) δ 5.52 (bs, 2H), 3.64–3.60 (2bs, 6H), 3.13–2.85 (4bs, 4H), 1.89 (2bs, 2H); ^13^C NMR (CDCl_3_) δ 172.9, 172.5, 131.9, 131.1, 130.5, 51.7, 51.6, 51.5, 51.4, 51.1, 44.5, 39.2, 38.8, 38.1; FTIR (ATR, cm^−1^) 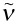: 3020 (w), 2951 (w), 1726 (s), 1434 (m), 1386 (w), 1347 (w), 1194 (m), 1169 (m), 1153 (m), 1095 (w), 1041 (w), 978 (w), 957 (w), 807 (w), 747 (m), 666 (w), 603 (w).

**Poly-M3:****^1^H NMR (CDCl_3_) δ 5.74 (bs, 1H), 5.56 (bs, 1H), 4.59–4.49 (2bs, 1H), 4.17–4.11 (2bs, 5H), 2.39 (bs, 2H), 2.04–2.02 (2s, 6H); ^13^C NMR (CDCl_3_) δ 170.8, 170.7, 133.3, 132.9, 132.4, 131.8, 81.5, 81.2, 62.0, 61.9, 61.8, 46.5, 46.2, 46.1, 45.9, 45.6, 21.0, 20.9; FTIR (ATR, cm^−1^) 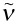: 3017 (w), 2958 (w), 2902 (w), 1733 (s), 1468 (w), 1434 (w), 1389 (w), 1366 (m), 1221 (s), 1119 (w), 1030 (s), 968 (m), 834 (w), 750 (m), 667 (w), 604 (m), 506 (w), 471 (w).

**Poly-M4:****^1^H NMR (CDCl_3_) δ 5.27–5.17 (2bs, 2H), 3.40–3.35 (2bs, 8H), 2.69 (bs, 1H), 2.32 (bs, 1H), 1.96 (bs, 3H), 1.55 (bs, 4H), 1.32–1.13 (2bs, 10H), 0.90 (bs, 6H); ^13^C NMR (CDCl_3_) δ 134.0, 133.8, 71.3, 71.2, 70.8, 70.6, 48.1, 47.8, 47.6, 47.0, 45.6, 45.3, 41.2, 40.1, 29.7, 28.7, 22.7, 14.2; FTIR (ATR, cm^−1^) 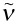: 3005 (w), 2919 (s), 2850 (s), 1465 (m), 1438 (w), 1261 (w), 1091 (w), 1071 (w), 1026 (w), 965 (s), 805 (w), 720 (w).

**Poly-M6:****^1^H NMR (CDCl_3_) δ 5.43–5.31 (m, 2H), 2.02–1.97 (m, 4H), 1.33–1.29 (bs, 8H); ^13^C NMR (CDCl_3_) δ 130.5, 130.0, 32.8, 29.9, 29.8, 29.4, 29.3, 29.2, 27.4; FTIR (ATR, cm^−1^) 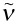: 2954 (m), 2929 (m), 2853 (m), 1795 (w), 1482 (w), 1465 (w), 1367 (w), 1104 (s), 1066 (m), 1010 (w), 966 (w), 741 (m).

## Supporting Information

File 1 Analytical data for **Ru-2**, the polymers prepared, details on the single crystal X-ray structural analysis of **Ru-2**, results for biphasic RCM.
