# Supplementary material for: Grubbs–Hoveyda type catalysts bearing a dicationic N-heterocyclic carbene for biphasic olefin metathesis reactions in ionic liquids
Source: Beilstein J Org Chem. 2015 Sep 15;11:1632–8. doi: 10.3762/bjoc.11.178 (PMC4660883; doi:10.3762/bjoc.11.178)
Supplement: File 1 — Analytical data for Ru-2, the polymers prepared, details on the single crystal X-ray structural analysis of Ru-2, results for biphasic RCM. [file Beilstein_J_Org_Chem-11-1632-s001.pdf]

Supporting Information

for

Grubbs–Hoveyda type catalysts bearing a dicationic *N*-heterocyclic carbene for biphasic olefin metathesis reactions in ionic liquids

Maximilian Koy[1], Hagen J. Altmann[1], Benjamin Autenrieth[1], Wolfgang Frey[2] and Michael R. Buchmeiser[1,3*]

Address: [1]Lehrstuhl für Makromolekulare Stoffe und Faserchemie, Institut für Polymerchemie, Universität Stuttgart, Pfaffenwaldring 55, D-70569 Stuttgart, Germany, Fax: +49 (0)711-685-64050, [2]Institut für Organische Chemie, Universität Stuttgart, Pfaffenwaldring 55, D-70569 Stuttgart, Germany and [3]Institut für Textilchemie und Chemiefaser (ITCF) Denkendorf, Körschtalstr. 26, D-73770 Denkendorf, Germany

Email: Michael Buchmeiser - michael.buchmeiser@ipoc.uni-stuttgart.de

*Corresponding author

Analytical data for Ru-2, the polymers prepared, details on the single crystal X-ray structural analysis of Ru-2, results for biphasic RCM

Table of Contents

1. Ring-closing metathesis (RCM) reactions under biphasic conditions S3
   1.1. General RCM procedure S3
   1.2. Results S3
2. References... S3
3. Analytical data for **Ru-2**... S5
4. Analytical data for polymers prepared by **Ru-2** S8
5. X-ray crystallography data for **Ru-2** S18

List of Figures

Figure S1: ^{1}H NMR of **Ru-2** in DMF-d_7. .... S5
Figure S2: ^{13}C NMR of **Ru-2** in DMF-d_7. .... S6
Figure S3: ^{19}F NMR of **Ru-2** in DMF-d_7. .... S7
Figure S4: ^{1}H NMR of poly-**M1** in $CDCl_3$. S8
Figure S5: ^{13}C NMR of poly-**M1** in $CDCl_3$. S9
Figure S6: ^{1}H NMR of poly-**M2** in $CDCl_3$. S10
Figure S7: ^{13}C NMR of poly-**M2** in $CDCl_3$. S11
Figure S8: ^{1}H NMR of poly-**M3** in $CDCl_3$. S12
Figure S9: ^{13}C NMR of poly-**M3** in $CDCl_3$. S13
Figure S10: ^{1}H NMR of poly-**M4** in $CDCl_3$. S14
Figure S11: ^{13}C NMR of poly-**M4** in $CDCl_3$. S15
Figure S12: ^{1}H NMR of poly-**M6** in $CDCl_3$. S16
Figure S13: ^{13}C NMR of poly-**M6** in $CDCl_3$. S17
Figure S14: Single crystal X-ray structure of **Ru-2**. Cosolvent and disordered triflates have been omitted for clarity. S18
Figure S15: Single crystal X-ray structure of **Ru-2** with DMF in the crystal lattice and disordered triflates. S19

1. Ring-closing metathesis (RCM) reactions under biphasic conditions

1.1. General RCM procedure

Ru-2 (3 mg, 3 µmol) and [BDMIM$^+$][BF$_4^-$] (250 mg) were placed inside a flame-dried Schlenk tube (25 mL) equipped with a magnetic stir bar. The reaction mixture was heated to the indicated temperature. To a separate Schlenk tube (10 mL) was added substrate (300 µmol), toluene (2 mL) and *tert*-butylbenzene (approx. 7 µL, standard for GC–-MS). An aliquote (approx. 5 µL) was removed and subjected to GC–MS analysis. The substrate solution was added via a syringe in one portion to the catalyst solution and the reaction mixture was allowed to stir at 70 °C for 14 h. After cooling to room temperature, ethyl vinyl ether (1 mL) was added and the reaction mixture was allowed to stir for another 30 min. The IL phase was extracted with diethyl ether. The combined organic phases were subjected to GC–MS analysis.

1.2. Results

Table S1: RCM reactions under biphasic conditions.[a]

Substrate	TON[b]
Diethyl diallylmalonate	55
1,7-Octadiene	70
Diallyl thioether	31
N,*N*-Diallyltosylamine[c]	58

[a] **Ru-2**, toluene, [BDMIM$^+$][BF$_4^-$], 70 °C, 14 h; [b] determined by comparing the ratios of product/standard (*tert*-butylbenzene) by GC–MS before and after the reaction; [c] substrate was prepared according to the literature [1-3].

2. References

1. Engle, K. M.; Lu, G.; Luo, S.-X.; Henling, L. M.; Takase, M. K.; Liu, P.; Houk, K. N.; Grubbs, R. *J. Am. Chem. Soc.*, **2015**, *137*, 5782-5792.

2. Gilbert, B. C.; Kalz, W.; Lindsay, C. I.; McGrail, P. T.; Parsons, A. F.; Whittaker, D. T. E. *J. Chem. Soc., Perkin Trans. 1* **2000**, 1187–1194.
3. So, C. M.; Kume, S.; Hayashi, T. *J. Am. Chem. Soc.* **2013**, *135*, 10990–10993.

3. Analytical data for Ru-2

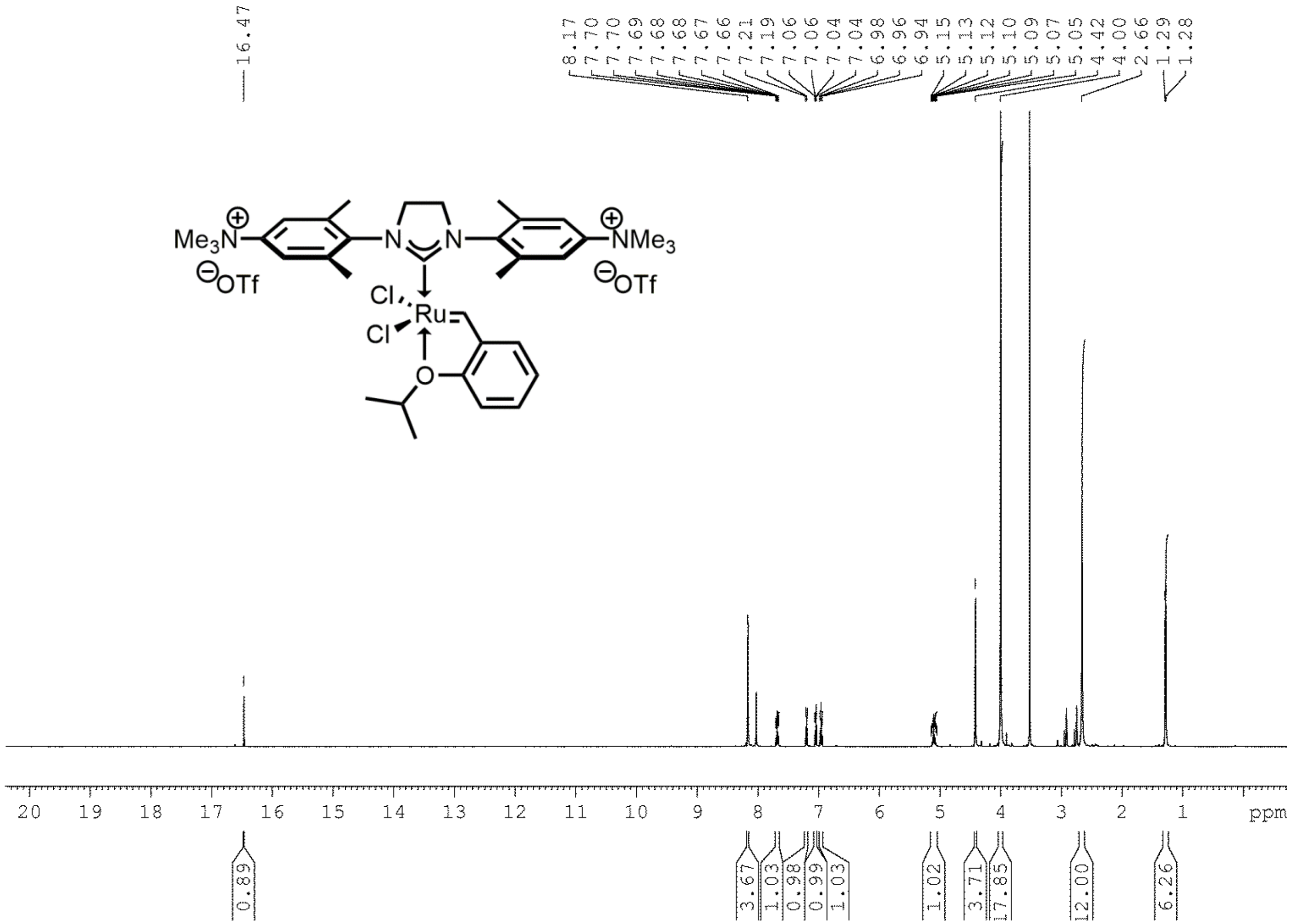

Figure S1: ^{1}H NMR of **Ru-2** in DMF-d_7.

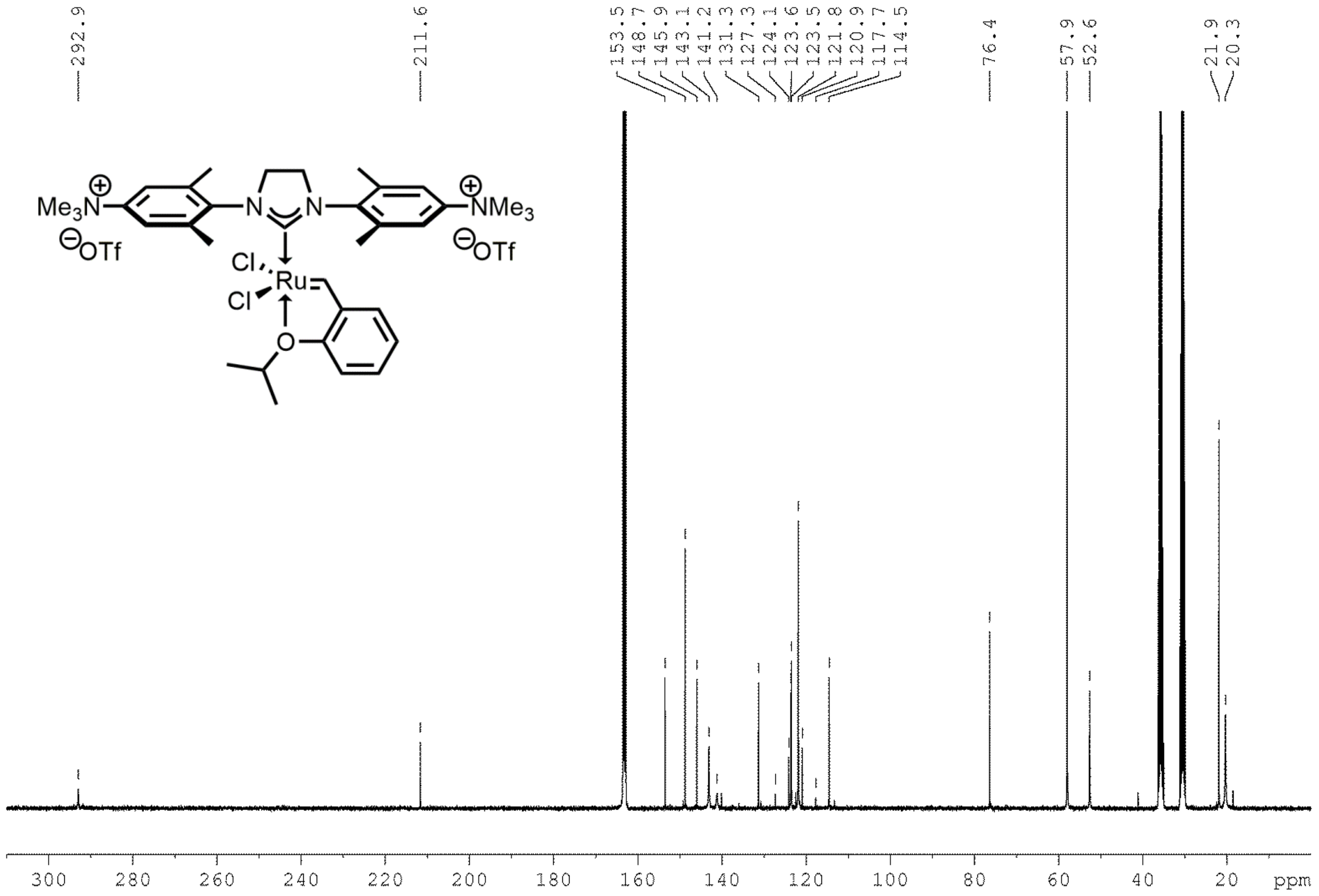

Figure S2: ^{13}C NMR of **Ru-2** in DMF-d_7.

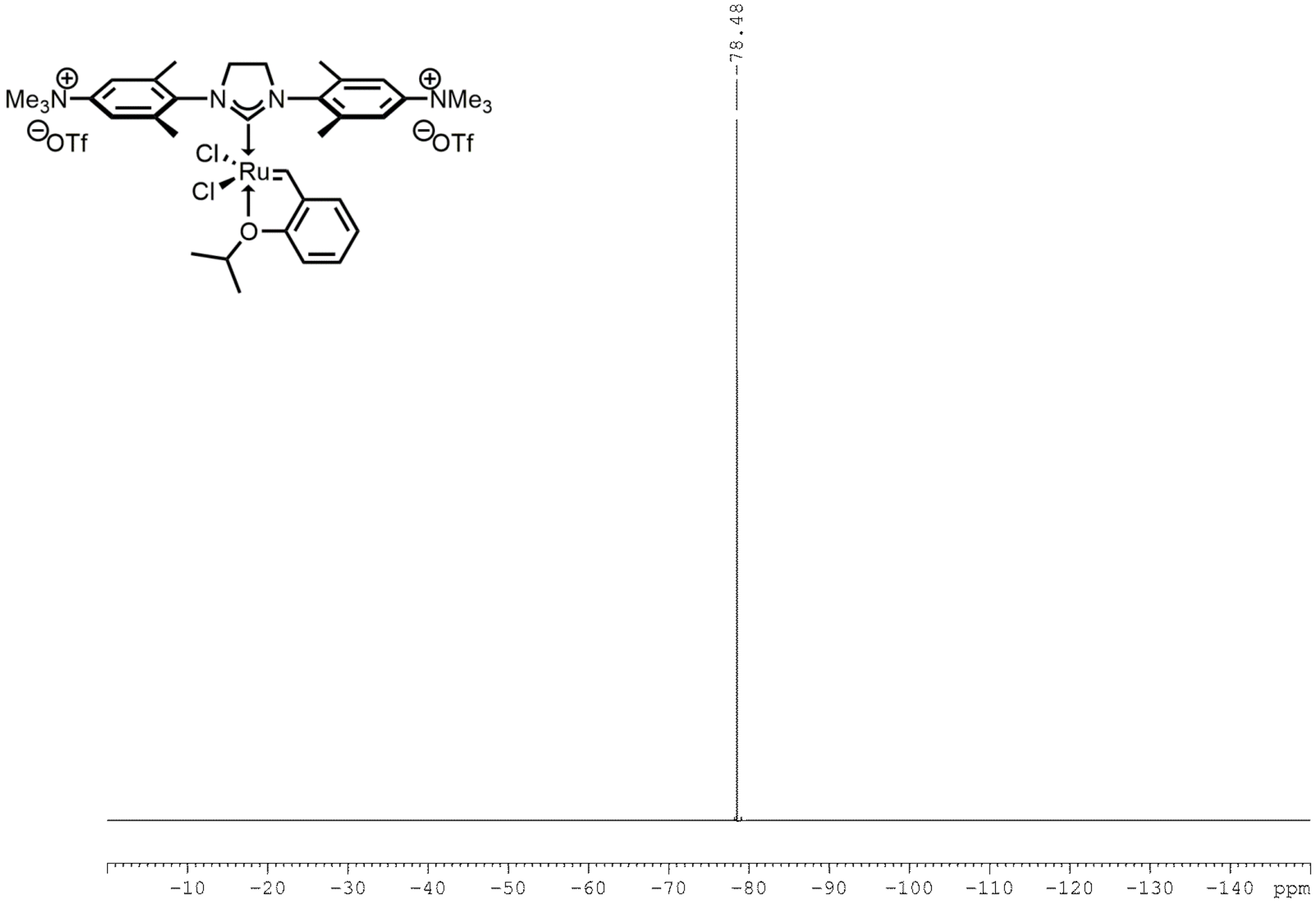

Figure S3: ^{19}F NMR of **Ru-2** in DMF-d_7.

4. Analytical data for polymers prepared by Ru-2

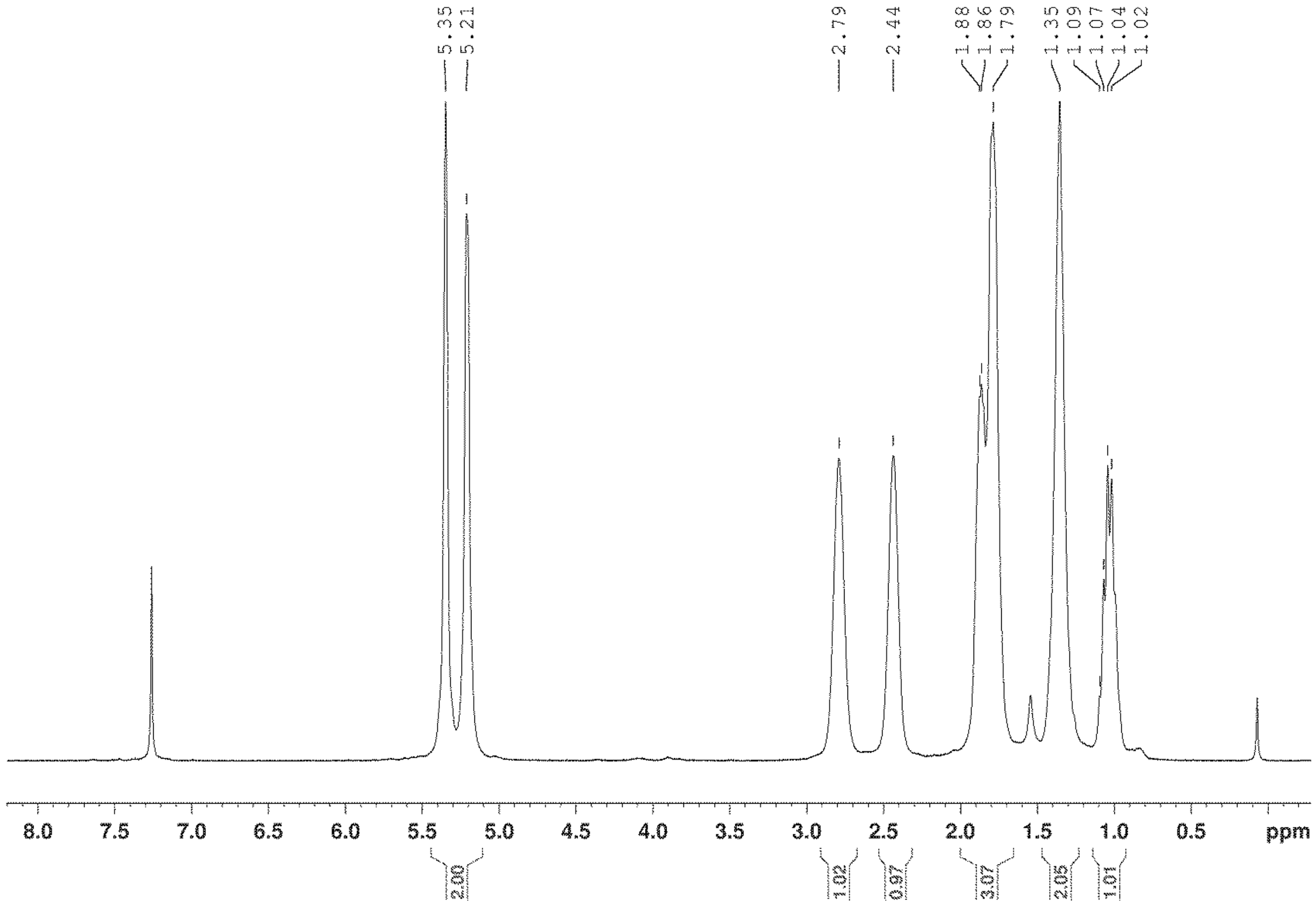

Figure S4: ^{1}H NMR of poly-**M1** in $CDCl_3$.

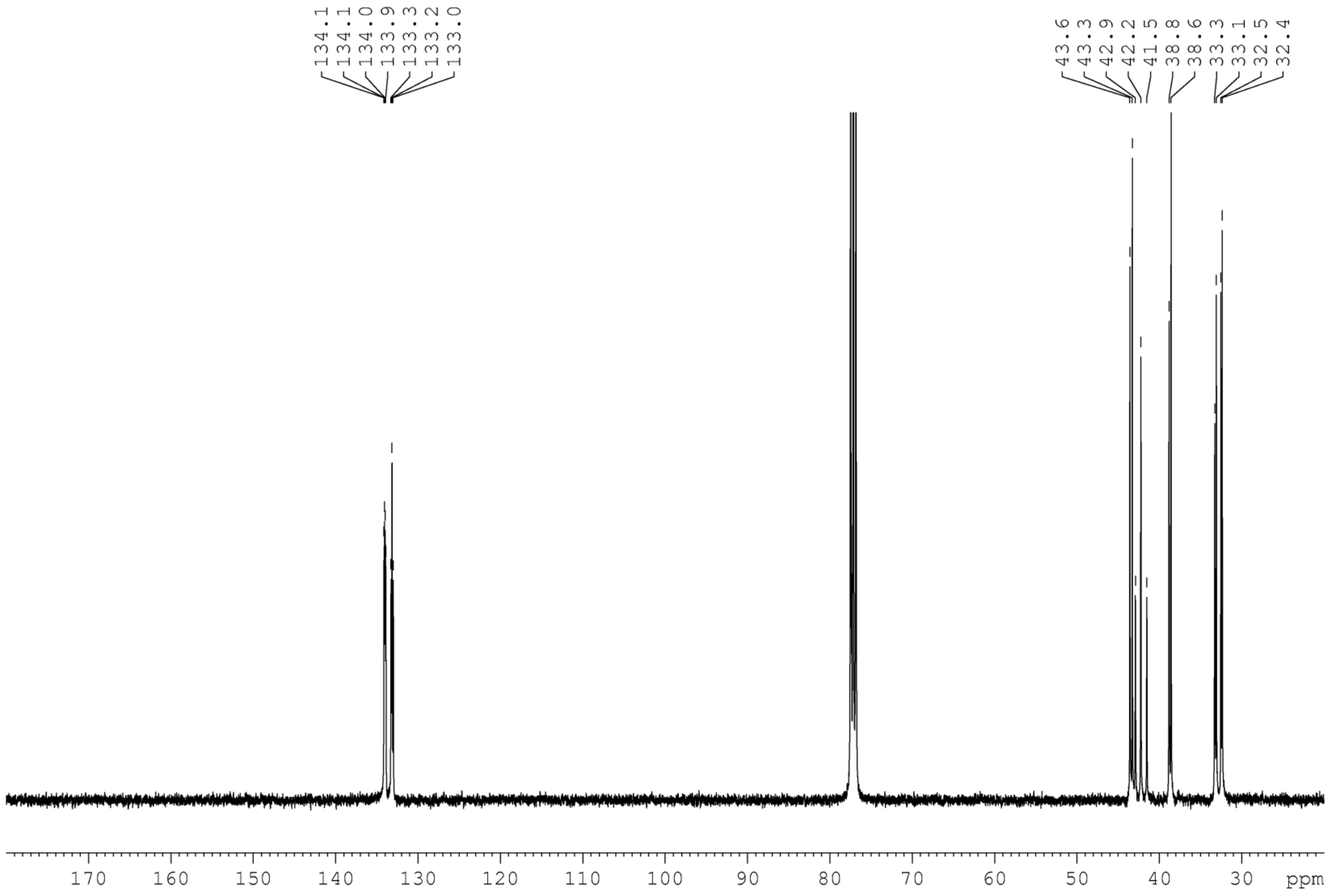

Figure S5: ^{13}C NMR of poly-**M1** in $CDCl_3$.

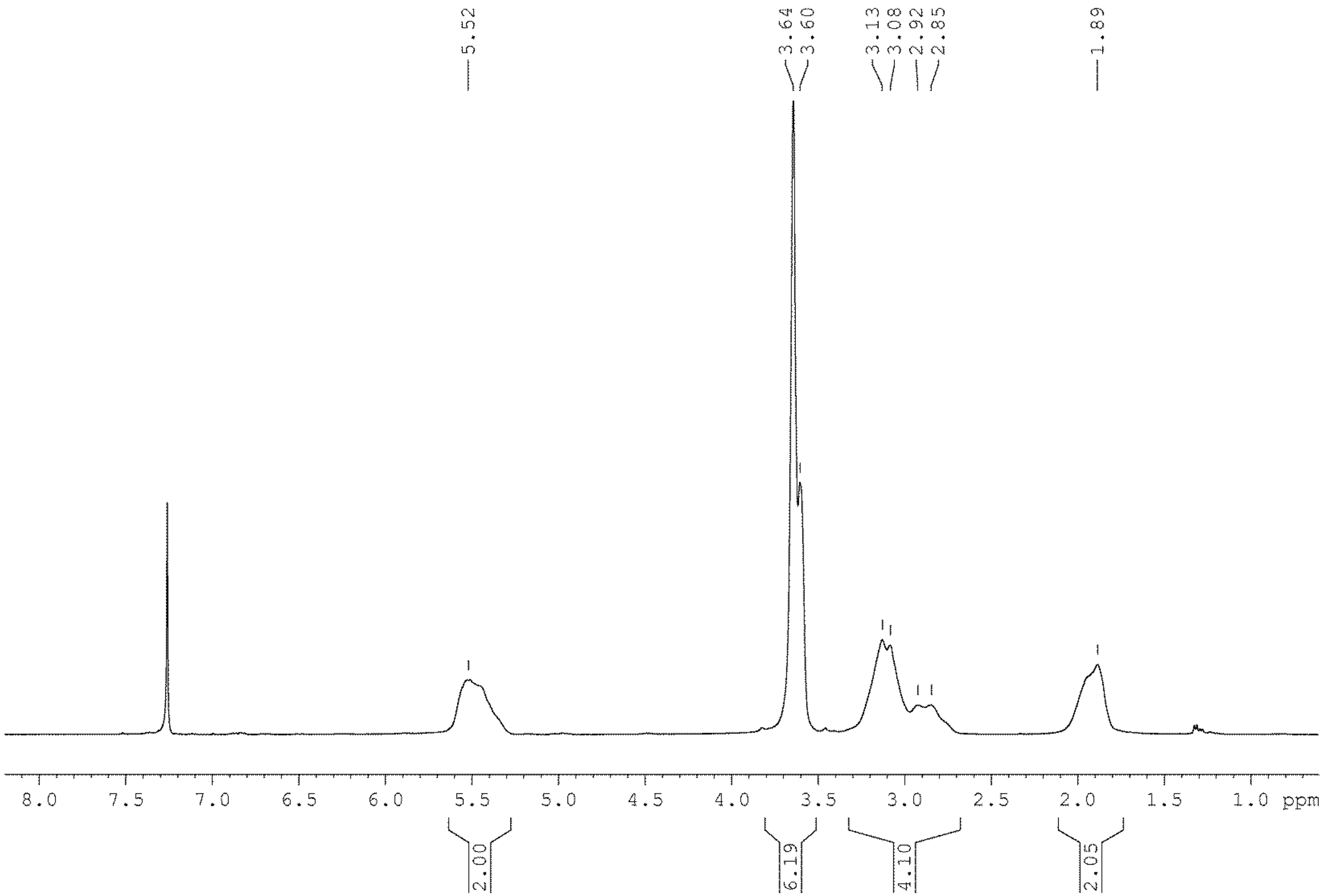

Figure S6: ^{1}H NMR of poly-**M2** in $CDCl_3$.

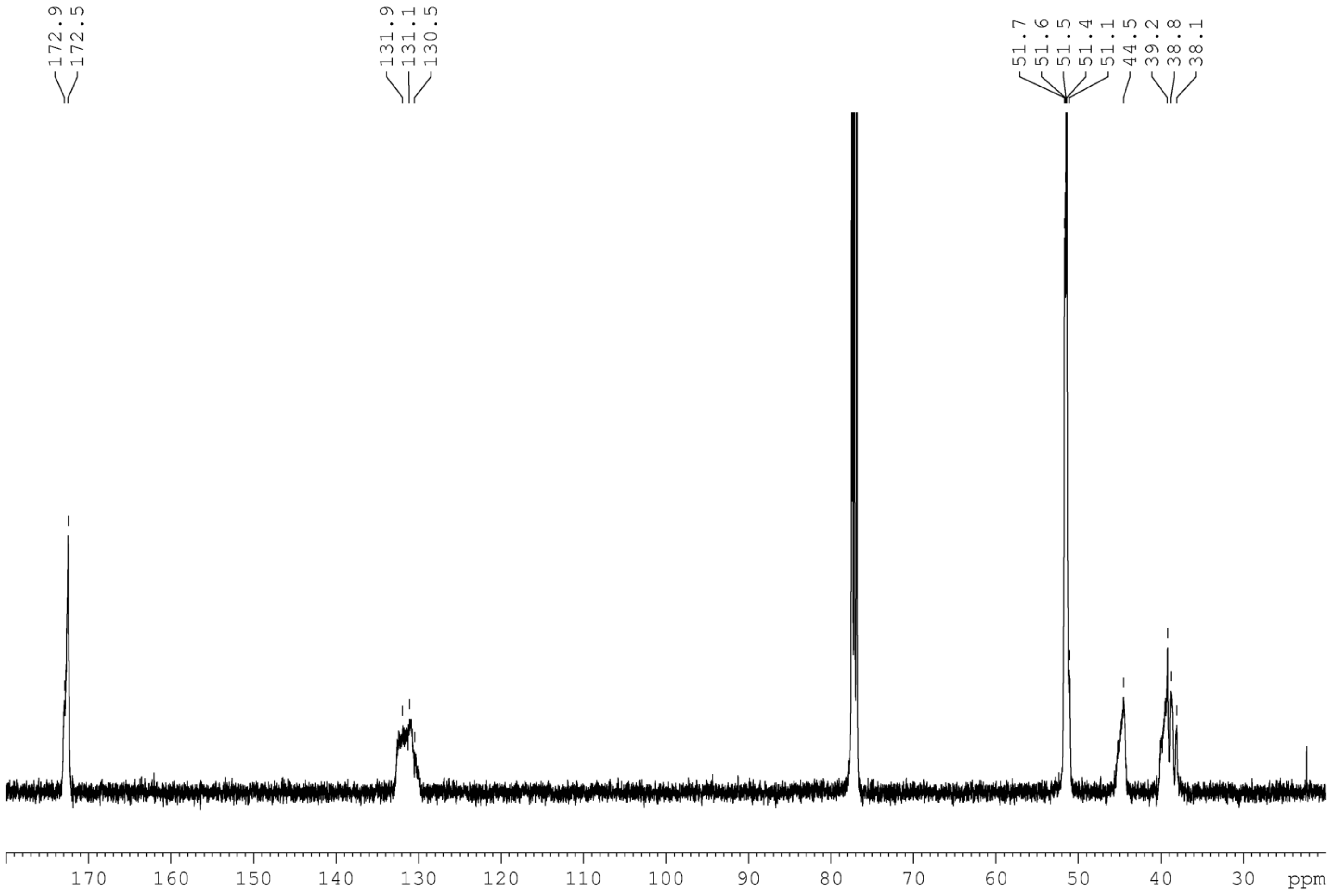

Figure S7: ^{13}C NMR of poly-**M2** in $CDCl_3$.

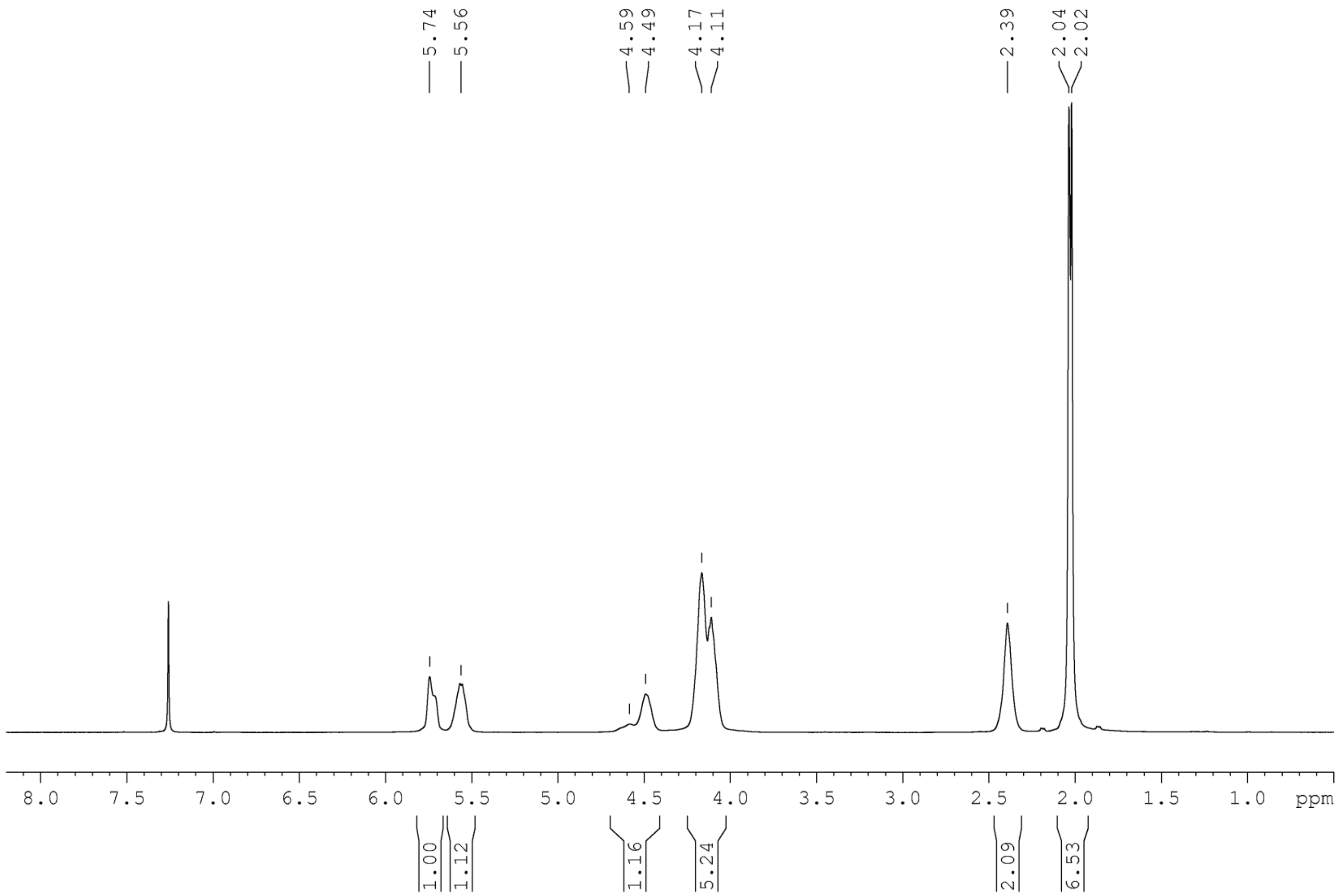

Figure S8: ^{1}H NMR of poly-**M3** in $CDCl_3$.

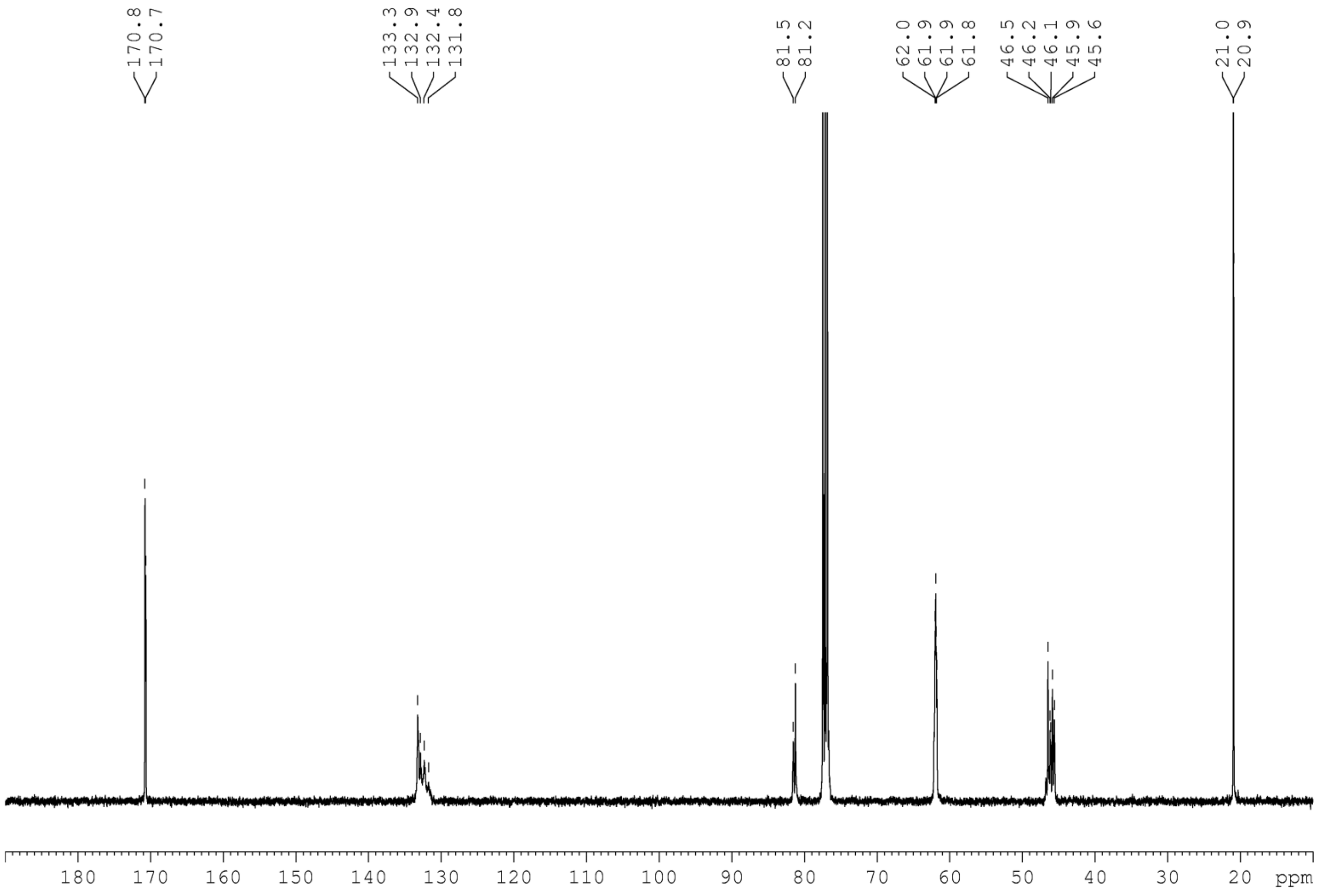

Figure S9: ^{13}C NMR of poly-**M3** in $CDCl_3$.

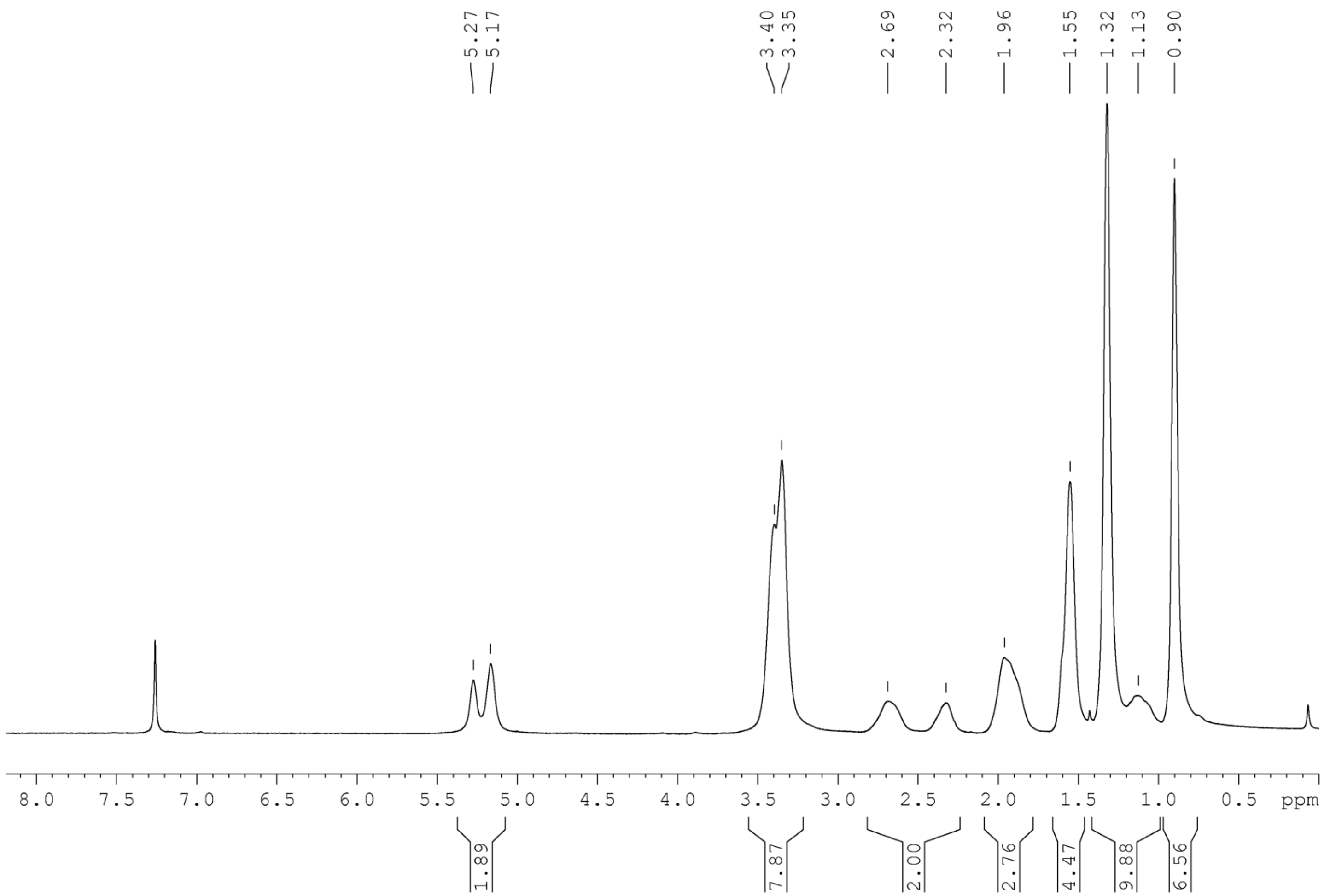

Figure S10: ^{1}H NMR of poly-**M4** in $CDCl_3$.

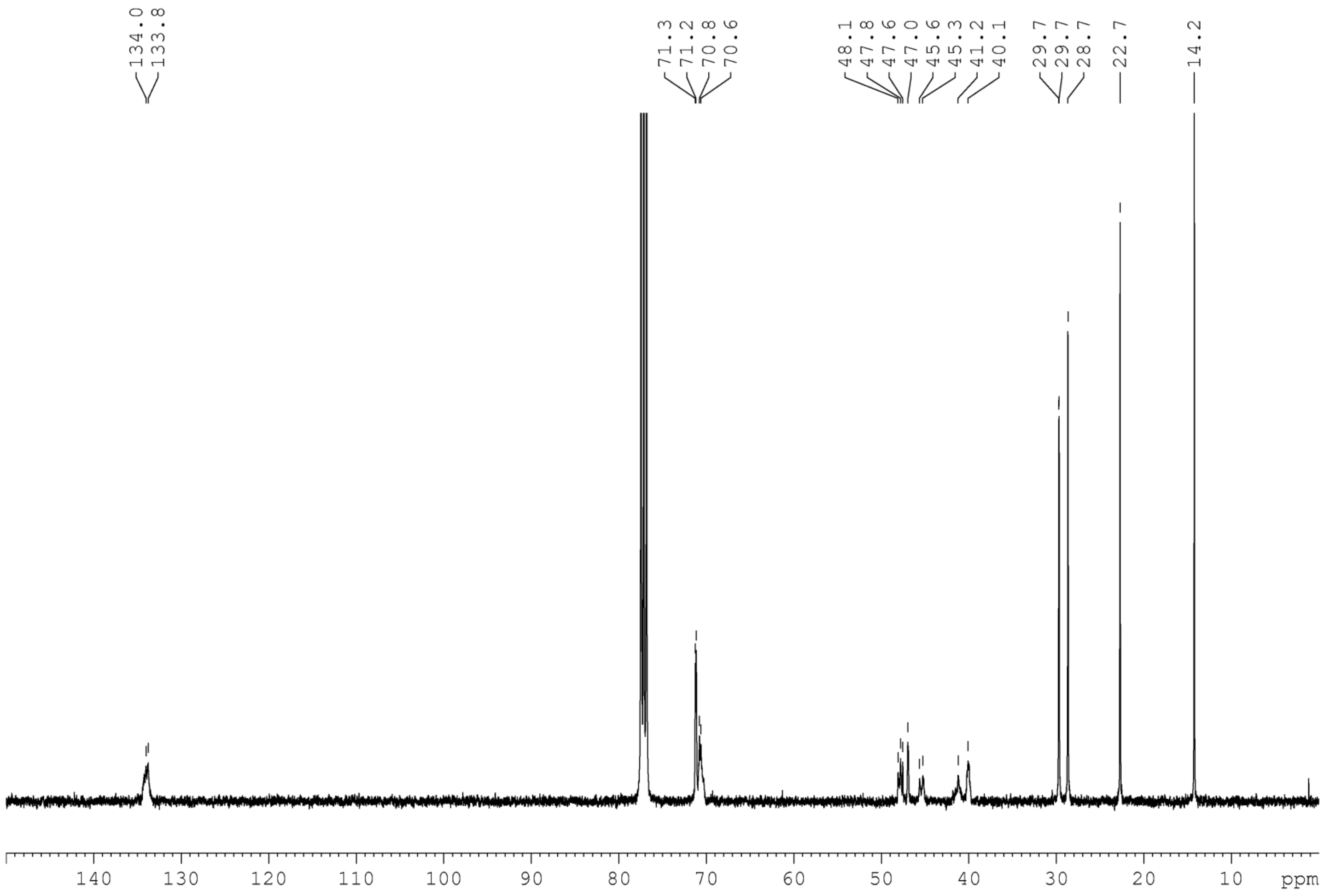

Figure S11: ^{13}C NMR of poly-**M4** in $CDCl_3$.

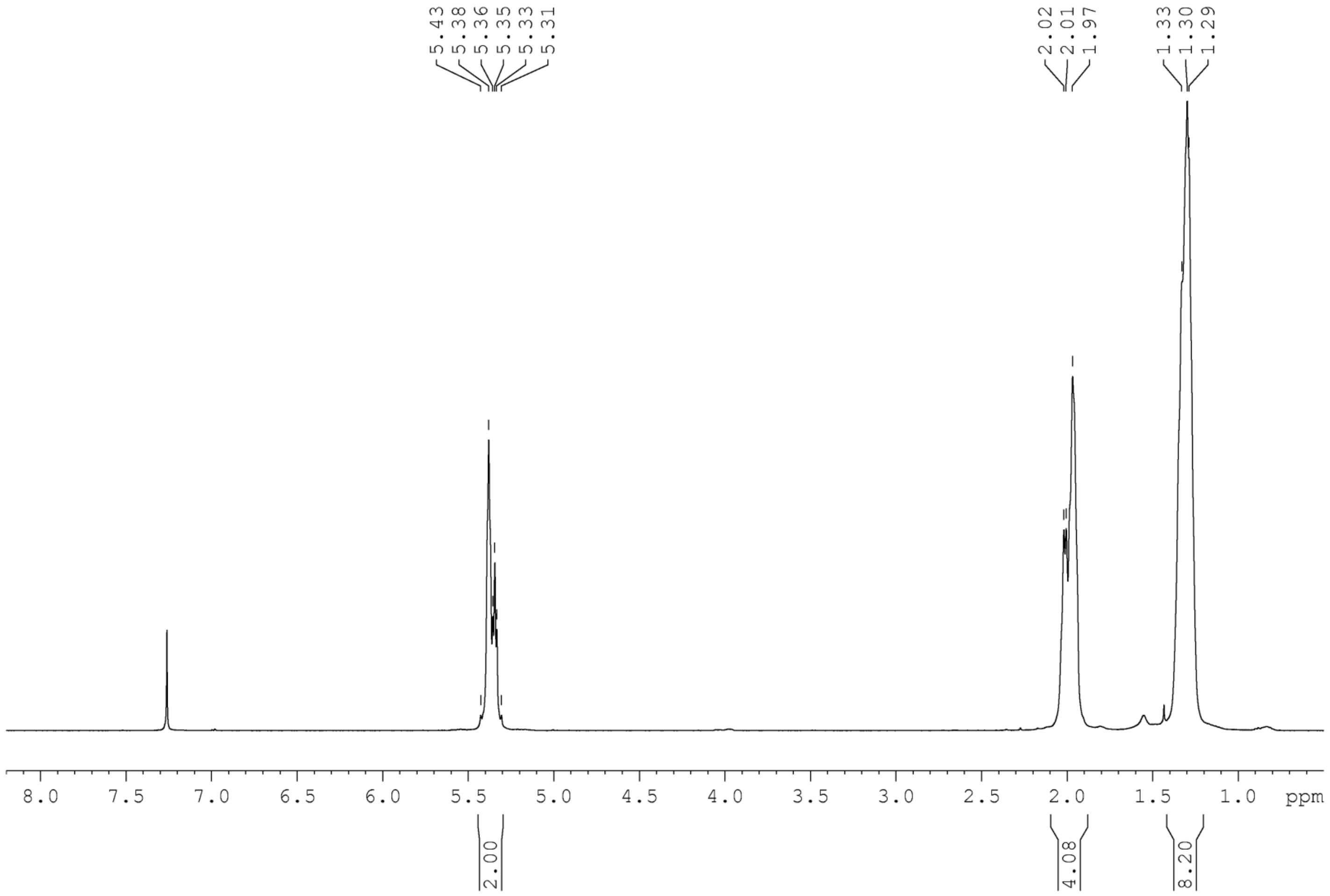

Figure S12: ^{1}H NMR of poly-**M6** in $CDCl_3$.

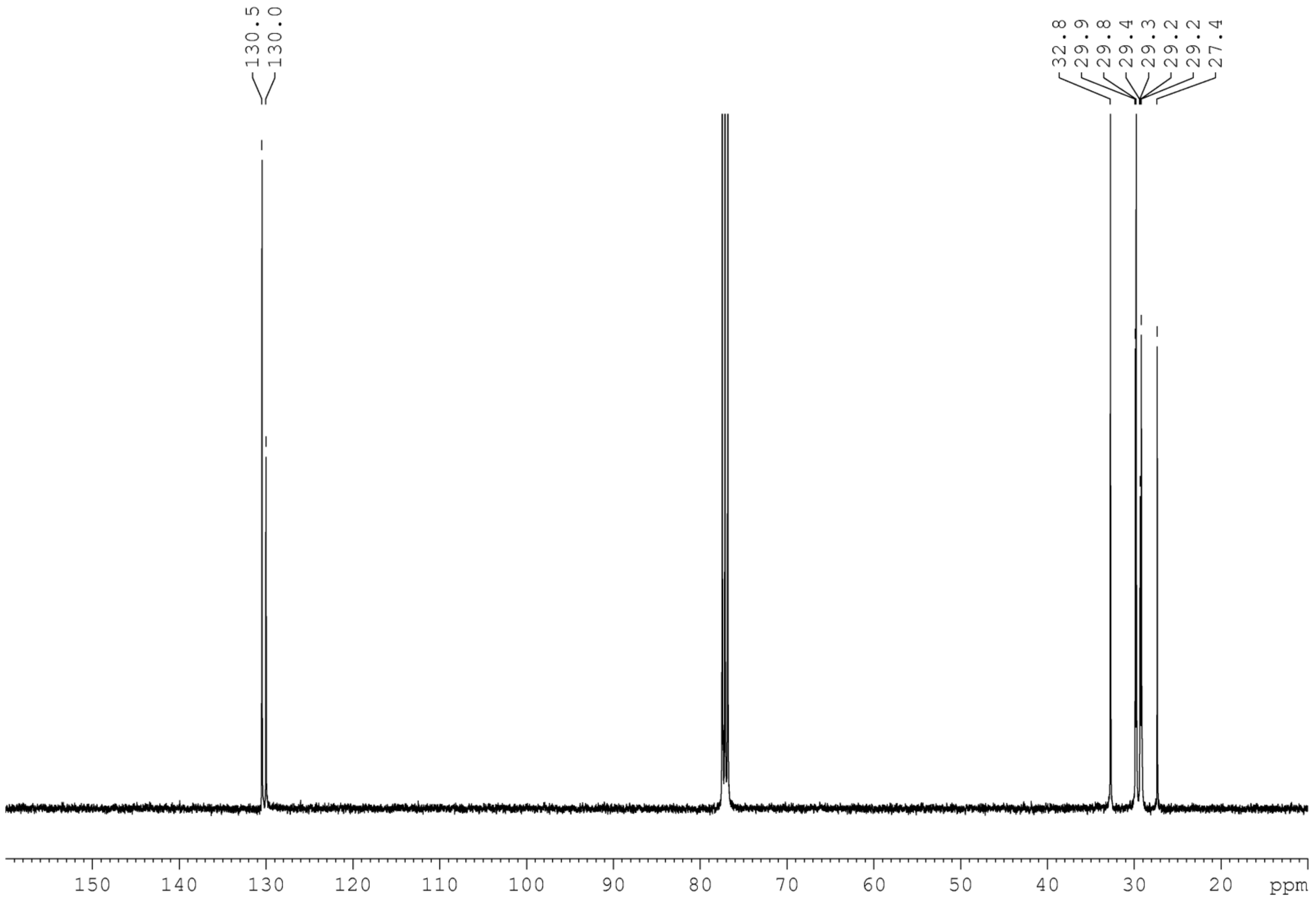

Figure S13: ^{13}C NMR of poly-**M6** in $CDCl_3$.

5. X-ray crystallography data for Ru-2

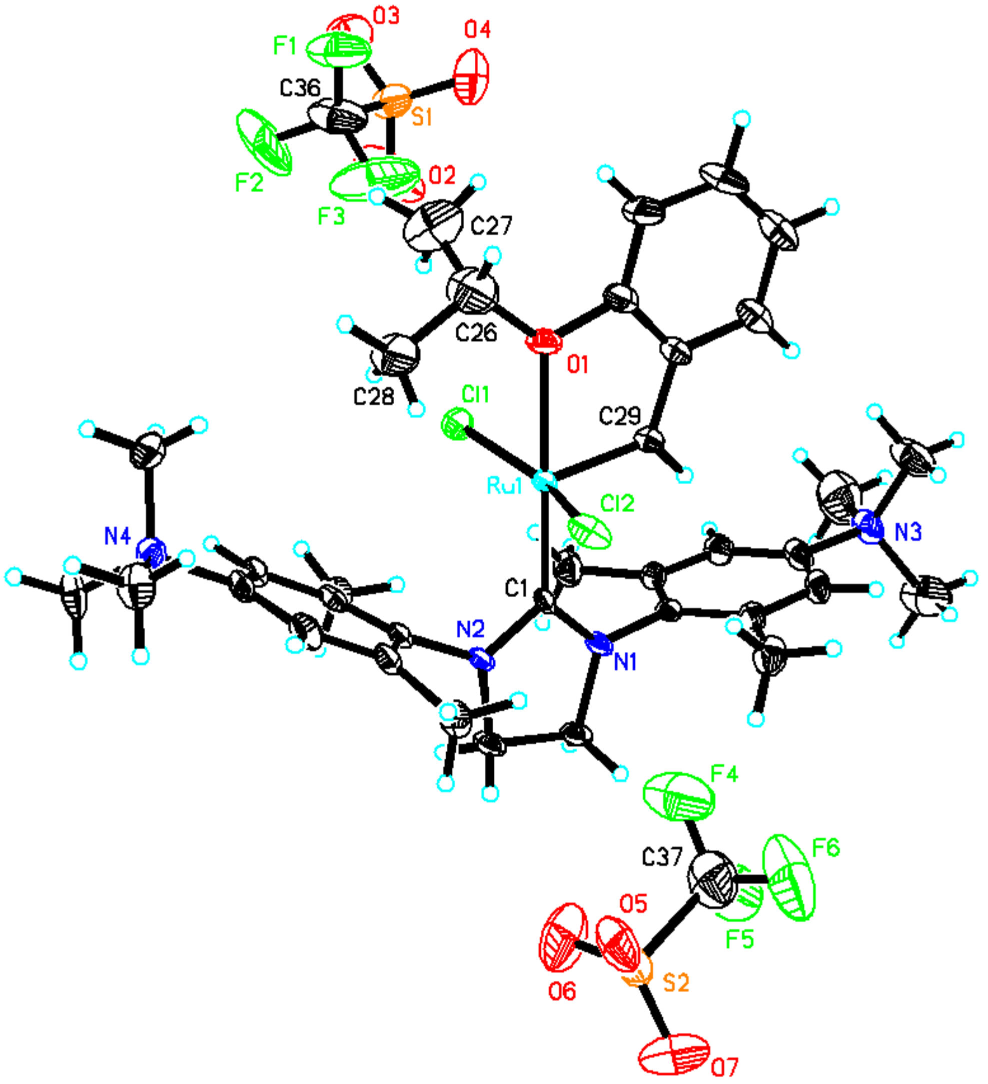

Figure S14: Single crystal X-ray structure of **Ru-2**. Cosolvent and disordered triflates have been omitted for clarity.

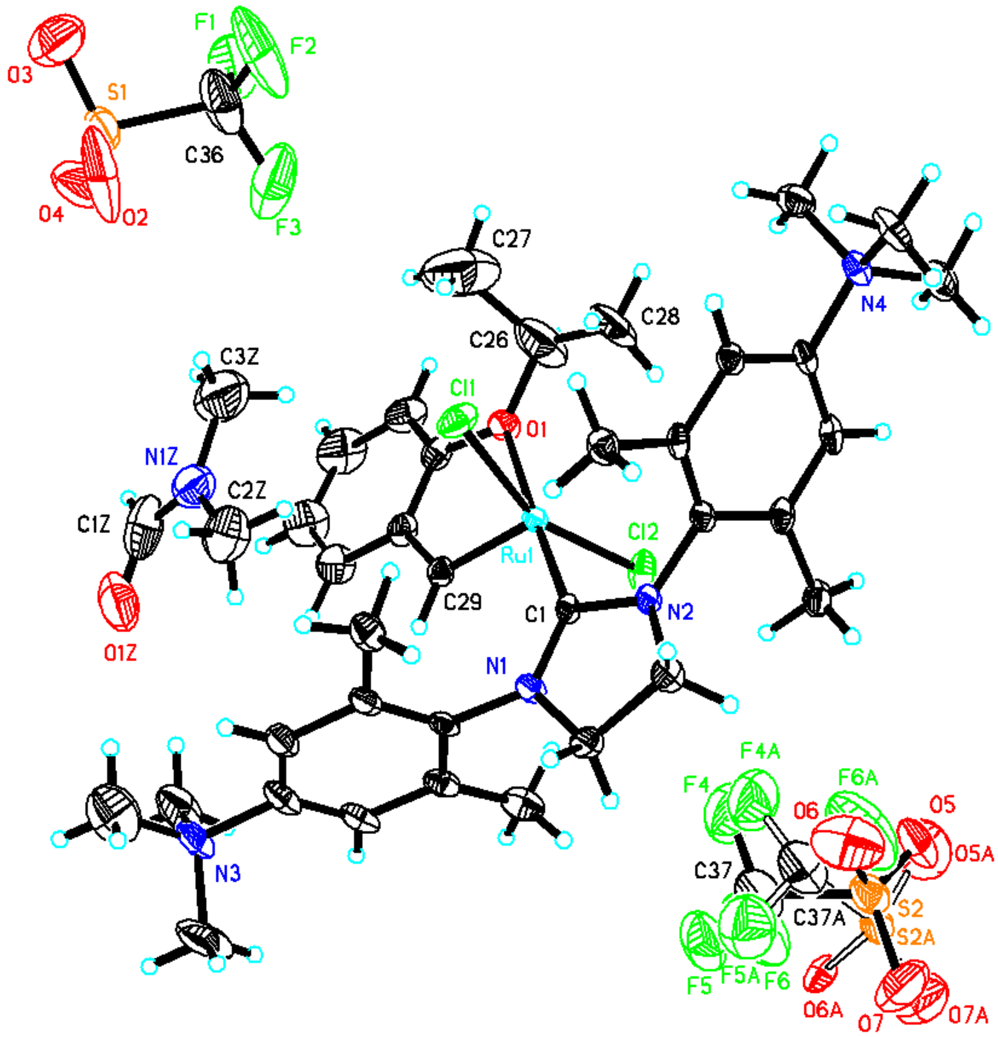

Figure S15: Single crystal X-ray structure of **Ru-2** with DMF in the crystal lattice and disordered triflates.

Table S2: Crystal data and structure refinement.

Empirical formula	C40 H57 Cl2 F6 N5 O8 Ru S2	
Formula weight	1086.00	
Temperature	100(2) K	
Wavelength	0.71073 Å	
Crystal system, space group	Triclinic, P -1	
Unit cell dimensions	a = 13.9808(6) Å	α = 106.079(3)°
	b = 13.9941(7) Å	β = 112.209(3)°
	c = 17.5026(13) Å	γ = 99.300(2)°
Volume	2906.6(3) Å^3	
Z, Calculated density	2, 1.241 Mg/m^3	
Absorption coefficient	0.497 mm^{-1}	
F(000)	1120	
Crystal size	0.57 x 0.19 x 0.17 mm	
Theta range for data collection	1.65 to 27.00 deg.	
Limiting indices	-17 ≤ h ≤ 17, -17 ≤ k ≤ 17, -22 ≤ l ≤ 22	
Reflections collected / unique	48136 / 12684 [R(int) = 0.0540]	
Completeness to theta = 27.00	99.8 %	
Absorption correction	Semi-empirical from equivalents	
Max. and min. transmission	0.7457 and 0.6795	
Refinement method	Full-matrix least-squares on F^2	
Data / restraints / parameters	12684 / 114 / 645	
Goodness-of-fit on F^2	1.033	
Final R indices [I>2σ(I)]	R1 = 0.0646, wR2 = 0.1508	
R indices (all data)	R1 = 0.0875, wR2 = 0.1614	
Largest diff. peak and hole	3.073 and -1.562 e·Å^{-3}	

****

REMARK: Disordered solvent density (DMF) squeezed bey PLATON !

****

Table S3: Atomic coordinates (x 10^4) and equivalent isotropic displacement parameters (Å^2 x 10^3) U(eq) is defined as one third of the trace of the orthogonalized U_{ij} tensor.

	x	y	z	U(eq)
Ru(1)	4647(1)	884(1)	2587(1)	16(1)
Cl(1)	5718(1)	335(1)	3672(1)	32(1)
Cl(2)	3094(1)	1022(1)	1535(1)	28(1)
O(1)	4208(3)	-754(2)	1633(2)	26(1)
C(1)	4981(3)	2326(3)	3403(3)	15(1)
N(1)	5703(3)	3239(3)	3626(2)	20(1)
N(2)	4365(3)	2545(3)	3837(2)	17(1)
C(2)	5585(4)	4149(3)	4216(3)	23(1)
N(3)	9253(3)	4071(3)	2826(3)	33(1)
C(3)	4700(4)	3643(3)	4419(3)	25(1)
N(4)	1377(3)	-608(3)	3970(3)	23(1)
C(4)	6586(3)	3404(3)	3405(3)	20(1)
C(5)	7575(4)	3344(3)	3957(3)	22(1)
C(6)	8452(4)	3568(3)	3770(3)	26(1)
C(7)	8307(4)	3822(3)	3033(3)	28(1)
C(8)	7322(4)	3876(3)	2479(3)	26(1)
C(9)	6434(4)	3676(3)	2663(3)	24(1)
C(10)	7711(4)	3032(4)	4740(3)	32(1)
C(11)	5368(4)	3739(4)	2085(3)	33(1)
C(12)	9018(5)	3309(5)	1948(4)	54(2)
C(13)	9424(7)	5142(5)	2796(7)	81(3)
C(14)	10274(6)	4008(7)	3479(5)	72(2)
C(15)	3602(3)	1765(3)	3878(3)	18(1)
C(16)	3983(3)	1299(3)	4497(3)	19(1)
C(17)	3232(3)	508(3)	4497(3)	20(1)
C(18)	2141(3)	248(3)	3935(3)	20(1)
C(19)	1764(3)	776(4)	3386(3)	23(1)

C(20)	2493(3)	1571(3)	3360(3)	21(1)
C(21)	5131(3)	1677(4)	5211(3)	24(1)
C(22)	2065(4)	2219(4)	2834(3)	27(1)
C(23)	1587(4)	-1623(4)	3632(4)	36(1)
C(24)	217(4)	-717(4)	3423(3)	32(1)
C(25)	1531(4)	-374(4)	4910(3)	33(1)
C(26)	3493(7)	-1719(5)	1515(6)	73(2)
C(27)	4454(10)	-2273(7)	2084(8)	113(4)
C(28)	2980(6)	-1443(5)	2118(6)	67(2)
C(29)	5557(3)	1067(3)	2080(3)	20(1)
C(30)	5528(4)	172(4)	1404(3)	25(1)
C(31)	6180(5)	247(4)	970(4)	39(1)
C(32)	6094(6)	-636(5)	314(4)	54(2)
C(33)	5364(6)	-1574(5)	88(4)	54(2)
C(34)	4711(5)	-1674(4)	505(3)	37(1)
C(35)	4802(4)	-796(4)	1160(3)	25(1)
S(1)	8892(1)	-2741(1)	4440(1)	43(1)
O(2)	9293(3)	-1792(5)	5098(4)	97(2)
O(3)	9213(4)	-3572(4)	4674(4)	70(1)
O(4)	8943(4)	-2713(4)	3641(4)	75(2)
C(36)	7459(5)	-3095(5)	4124(6)	69(2)
F(1)	6936(3)	-3949(3)	3450(3)	77(1)
F(2)	7219(4)	-3119(4)	4756(3)	106(2)
F(3)	7043(3)	-2330(4)	3893(4)	113(2)
S(2)	2423(1)	5445(1)	2874(1)	36(1)
O(5)	1382(4)	4682(4)	2214(4)	49(2)
O(6)	2954(6)	5220(6)	3653(4)	74(2)
O(7)	2440(6)	6499(4)	3007(5)	69(2)
C(37)	3301(5)	5132(6)	2396(5)	62(2)
F(4)	3281(6)	4150(5)	2241(5)	86(2)
F(5)	4299(3)	5779(4)	2930(4)	69(1)
F(6)	3002(6)	5169(6)	1606(4)	111(2)
S(2A)	2037(6)	5501(6)	2189(5)	33(2)

O(5A)	1130(30)	4590(30)	1840(30)	89(16)
O(6A)	2428(15)	5727(15)	1580(11)	27(4)
O(7A)	2100(30)	6525(15)	2680(20)	67(11)
C(37A)	3066(15)	5113(18)	2926(14)	62(2)
F(4A)	3170(30)	4181(18)	2560(20)	86(2)
F(5A)	4033(19)	5844(18)	3346(19)	80(8)
F(6A)	2710(40)	5060(30)	3540(30)	111(2)
N(1Z)	8138(4)	13(4)	2458(4)	48(1)
O(1Z)	9102(4)	942(5)	1979(4)	76(2)
C(1Z)	8586(6)	102(7)	1920(4)	62(2)
C(2Z)	8191(6)	955(6)	3111(5)	58(2)
C(3Z)	7557(7)	-981(6)	2417(7)	90(3)

Table S4: Bond lengths [Å] and angles [°].

Ru(1)-C(29)	1.828(4)
Ru(1)-C(1)	1.982(4)
Ru(1)-O(1)	2.252(3)
Ru(1)-Cl(2)	2.3350(12)
Ru(1)-Cl(1)	2.3389(12)
O(1)-C(35)	1.372(5)
O(1)-C(26)	1.455(7)
C(1)-N(1)	1.346(5)
C(1)-N(2)	1.365(5)
N(1)-C(4)	1.429(5)
N(1)-C(2)	1.477(5)
N(2)-C(15)	1.434(5)
N(2)-C(3)	1.469(5)
C(2)-C(3)	1.533(6)
C(2)-H(2A)	0.9900
C(2)-H(2B)	0.9900
N(3)-C(12)	1.484(7)
N(3)-C(14)	1.489(9)
N(3)-C(13)	1.497(7)
N(3)-C(7)	1.513(5)
C(3)-H(3A)	0.9900
C(3)-H(3B)	0.9900
N(4)-C(24)	1.496(6)
N(4)-C(18)	1.502(5)
N(4)-C(23)	1.502(6)
N(4)-C(25)	1.507(6)
C(4)-C(5)	1.388(7)
C(4)-C(9)	1.403(6)
C(5)-C(6)	1.395(6)
C(5)-C(10)	1.504(6)
C(6)-C(7)	1.382(7)

C(6)-H(6)	0.9500
C(7)-C(8)	1.381(7)
C(8)-C(9)	1.403(6)
C(8)-H(8)	0.9500
C(9)-C(11)	1.484(7)
C(10)-H(10A)	0.9800
C(10)-H(10B)	0.9800
C(10)-H(10C)	0.9800
C(11)-H(11A)	0.9800
C(11)-H(11B)	0.9800
C(11)-H(11C)	0.9800
C(12)-H(12A)	0.9800
C(12)-H(12B)	0.9800
C(12)-H(12C)	0.9800
C(13)-H(13A)	0.9800
C(13)-H(13B)	0.9800
C(13)-H(13C)	0.9800
C(14)-H(14A)	0.9800
C(14)-H(14B)	0.9800
C(14)-H(14C)	0.9800
C(15)-C(16)	1.397(6)
C(15)-C(20)	1.402(6)
C(16)-C(17)	1.398(6)
C(16)-C(21)	1.504(6)
C(17)-C(18)	1.384(6)
C(17)-H(17)	0.9500
C(18)-C(19)	1.372(6)
C(19)-C(20)	1.404(6)
C(19)-H(19)	0.9500
C(20)-C(22)	1.500(6)
C(21)-H(21A)	0.9800
C(21)-H(21B)	0.9800
C(21)-H(21C)	0.9800

C(22)-H(22A)	0.9800
C(22)-H(22B)	0.9800
C(22)-H(22C)	0.9800
C(23)-H(23A)	0.9800
C(23)-H(23B)	0.9800
C(23)-H(23C)	0.9800
C(24)-H(24A)	0.9800
C(24)-H(24B)	0.9800
C(24)-H(24C)	0.9800
C(25)-H(25A)	0.9800
C(25)-H(25B)	0.9800
C(25)-H(25C)	0.9800
C(26)-C(28)	1.487(9)
C(26)-C(27)	1.774(14)
C(26)-H(26)	1.0000
C(27)-H(27A)	0.9800
C(27)-H(27B)	0.9800
C(27)-H(27C)	0.9800
C(28)-H(28A)	0.9800
C(28)-H(28B)	0.9800
C(28)-H(28C)	0.9800
C(29)-C(30)	1.449(6)
C(29)-H(29)	0.9500
C(30)-C(31)	1.398(6)
C(30)-C(35)	1.399(6)
C(31)-C(32)	1.387(8)
C(31)-H(31)	0.9500
C(32)-C(33)	1.377(9)
C(32)-H(32)	0.9500
C(33)-C(34)	1.379(8)
C(33)-H(33)	0.9500
C(34)-C(35)	1.379(6)
C(34)-H(34)	0.9500

S(1)-O(2)	1.352(5)
S(1)-O(3)	1.419(5)
S(1)-O(4)	1.438(5)
S(1)-C(36)	1.804(6)
C(36)-F(2)	1.279(9)
C(36)-F(1)	1.280(7)
C(36)-F(3)	1.382(9)
S(2)-O(7)	1.424(5)
S(2)-O(6)	1.434(6)
S(2)-O(5)	1.461(5)
S(2)-C(37)	1.774(6)
C(37)-F(6)	1.303(7)
C(37)-F(4)	1.318(7)
C(37)-F(5)	1.333(7)
S(2A)-O(7A)	1.420(10)
S(2A)-O(5A)	1.434(10)
S(2A)-O(6A)	1.451(9)
S(2A)-C(37A)	1.808(10)
C(37A)-F(4A)	1.341(10)
C(37A)-F(5A)	1.341(10)
C(37A)-F(6A)	1.346(10)
N(1Z)-C(1Z)	1.335(9)
N(1Z)-C(2Z)	1.455(8)
N(1Z)-C(3Z)	1.462(9)
O(1Z)-C(1Z)	1.230(9)
C(1Z)-H(1Z)	0.9500
C(2Z)-H(2Z1)	0.9800
C(2Z)-H(2Z2)	0.9800
C(2Z)-H(2Z3)	0.9800
C(3Z)-H(3Z1)	0.9800
C(3Z)-H(3Z2)	0.9800
C(3Z)-H(3Z3)	0.9800

C(29)-Ru(1)-C(1)	101.54(17)
C(29)-Ru(1)-O(1)	79.35(15)
C(1)-Ru(1)-O(1)	177.96(15)
C(29)-Ru(1)-Cl(2)	99.33(14)
C(1)-Ru(1)-Cl(2)	91.36(12)
O(1)-Ru(1)-Cl(2)	86.68(9)
C(29)-Ru(1)-Cl(1)	98.27(14)
C(1)-Ru(1)-Cl(1)	95.85(12)
O(1)-Ru(1)-Cl(1)	85.81(9)
Cl(2)-Ru(1)-Cl(1)	159.22(5)
C(35)-O(1)-C(26)	119.0(4)
C(35)-O(1)-Ru(1)	110.6(3)
C(26)-O(1)-Ru(1)	130.2(3)
N(1)-C(1)-N(2)	106.4(3)
N(1)-C(1)-Ru(1)	133.7(3)
N(2)-C(1)-Ru(1)	119.8(3)
C(1)-N(1)-C(4)	127.6(3)
C(1)-N(1)-C(2)	114.2(3)
C(4)-N(1)-C(2)	118.0(3)
C(1)-N(2)-C(15)	123.9(3)
C(1)-N(2)-C(3)	114.1(3)
C(15)-N(2)-C(3)	120.8(3)
N(1)-C(2)-C(3)	102.5(3)
N(1)-C(2)-H(2A)	111.3
C(3)-C(2)-H(2A)	111.3
N(1)-C(2)-H(2B)	111.3
C(3)-C(2)-H(2B)	111.3
H(2A)-C(2)-H(2B)	109.2
C(12)-N(3)-C(14)	106.3(5)
C(12)-N(3)-C(13)	109.0(5)
C(14)-N(3)-C(13)	109.5(6)
C(12)-N(3)-C(7)	109.8(4)
C(14)-N(3)-C(7)	113.1(4)

C(13)-N(3)-C(7)	109.0(4)
N(2)-C(3)-C(2)	102.3(3)
N(2)-C(3)-H(3A)	111.3
C(2)-C(3)-H(3A)	111.3
N(2)-C(3)-H(3B)	111.3
C(2)-C(3)-H(3B)	111.3
H(3A)-C(3)-H(3B)	109.2
C(24)-N(4)-C(18)	112.3(3)
C(24)-N(4)-C(23)	108.6(4)
C(18)-N(4)-C(23)	109.3(3)
C(24)-N(4)-C(25)	106.5(3)
C(18)-N(4)-C(25)	110.1(3)
C(23)-N(4)-C(25)	110.1(4)
C(5)-C(4)-C(9)	122.4(4)
C(5)-C(4)-N(1)	119.0(4)
C(9)-C(4)-N(1)	118.5(4)
C(4)-C(5)-C(6)	118.8(4)
C(4)-C(5)-C(10)	121.4(4)
C(6)-C(5)-C(10)	119.8(4)
C(7)-C(6)-C(5)	119.2(5)
C(7)-C(6)-H(6)	120.4
C(5)-C(6)-H(6)	120.4
C(8)-C(7)-C(6)	122.2(4)
C(8)-C(7)-N(3)	118.1(4)
C(6)-C(7)-N(3)	119.7(4)
C(7)-C(8)-C(9)	119.7(4)
C(7)-C(8)-H(8)	120.2
C(9)-C(8)-H(8)	120.2
C(8)-C(9)-C(4)	117.7(4)
C(8)-C(9)-C(11)	120.9(4)
C(4)-C(9)-C(11)	121.4(4)
C(5)-C(10)-H(10A)	109.5
C(5)-C(10)-H(10B)	109.5

H(10A)-C(10)-H(10B)	109.5
C(5)-C(10)-H(10C)	109.5
H(10A)-C(10)-H(10C)	109.5
H(10B)-C(10)-H(10C)	109.5
C(9)-C(11)-H(11A)	109.5
C(9)-C(11)-H(11B)	109.5
H(11A)-C(11)-H(11B)	109.5
C(9)-C(11)-H(11C)	109.5
H(11A)-C(11)-H(11C)	109.5
H(11B)-C(11)-H(11C)	109.5
N(3)-C(12)-H(12A)	109.5
N(3)-C(12)-H(12B)	109.5
H(12A)-C(12)-H(12B)	109.5
N(3)-C(12)-H(12C)	109.5
H(12A)-C(12)-H(12C)	109.5
H(12B)-C(12)-H(12C)	109.5
N(3)-C(13)-H(13A)	109.5
N(3)-C(13)-H(13B)	109.5
H(13A)-C(13)-H(13B)	109.5
N(3)-C(13)-H(13C)	109.5
H(13A)-C(13)-H(13C)	109.5
H(13B)-C(13)-H(13C)	109.5
N(3)-C(14)-H(14A)	109.5
N(3)-C(14)-H(14B)	109.5
H(14A)-C(14)-H(14B)	109.5
N(3)-C(14)-H(14C)	109.5
H(14A)-C(14)-H(14C)	109.5
H(14B)-C(14)-H(14C)	109.5
C(16)-C(15)-C(20)	121.9(4)
C(16)-C(15)-N(2)	118.9(4)
C(20)-C(15)-N(2)	119.0(4)
C(15)-C(16)-C(17)	117.8(4)
C(15)-C(16)-C(21)	123.0(4)

C(17)-C(16)-C(21)	118.9(4)
C(18)-C(17)-C(16)	120.4(4)
C(18)-C(17)-H(17)	119.8
C(16)-C(17)-H(17)	119.8
C(19)-C(18)-C(17)	121.2(4)
C(19)-C(18)-N(4)	121.1(4)
C(17)-C(18)-N(4)	117.7(4)
C(18)-C(19)-C(20)	120.1(4)
C(18)-C(19)-H(19)	119.9
C(20)-C(19)-H(19)	119.9
C(15)-C(20)-C(19)	117.9(4)
C(15)-C(20)-C(22)	122.5(4)
C(19)-C(20)-C(22)	119.5(4)
C(16)-C(21)-H(21A)	109.5
C(16)-C(21)-H(21B)	109.5
H(21A)-C(21)-H(21B)	109.5
C(16)-C(21)-H(21C)	109.5
H(21A)-C(21)-H(21C)	109.5
H(21B)-C(21)-H(21C)	109.5
C(20)-C(22)-H(22A)	109.5
C(20)-C(22)-H(22B)	109.5
H(22A)-C(22)-H(22B)	109.5
C(20)-C(22)-H(22C)	109.5
H(22A)-C(22)-H(22C)	109.5
H(22B)-C(22)-H(22C)	109.5
N(4)-C(23)-H(23A)	109.5
N(4)-C(23)-H(23B)	109.5
H(23A)-C(23)-H(23B)	109.5
N(4)-C(23)-H(23C)	109.5
H(23A)-C(23)-H(23C)	109.5
H(23B)-C(23)-H(23C)	109.5
N(4)-C(24)-H(24A)	109.5
N(4)-C(24)-H(24B)	109.5

H(24A)-C(24)-H(24B)	109.5
N(4)-C(24)-H(24C)	109.5
H(24A)-C(24)-H(24C)	109.5
H(24B)-C(24)-H(24C)	109.5
N(4)-C(25)-H(25A)	109.5
N(4)-C(25)-H(25B)	109.5
H(25A)-C(25)-H(25B)	109.5
N(4)-C(25)-H(25C)	109.5
H(25A)-C(25)-H(25C)	109.5
H(25B)-C(25)-H(25C)	109.5
O(1)-C(26)-C(28)	107.3(5)
O(1)-C(26)-C(27)	100.6(6)
C(28)-C(26)-C(27)	101.7(7)
O(1)-C(26)-H(26)	115.2
C(28)-C(26)-H(26)	115.2
C(27)-C(26)-H(26)	115.2
C(26)-C(27)-H(27A)	109.5
C(26)-C(27)-H(27B)	109.5
H(27A)-C(27)-H(27B)	109.5
C(26)-C(27)-H(27C)	109.5
H(27A)-C(27)-H(27C)	109.5
H(27B)-C(27)-H(27C)	109.5
C(26)-C(28)-H(28A)	109.5
C(26)-C(28)-H(28B)	109.5
H(28A)-C(28)-H(28B)	109.5
C(26)-C(28)-H(28C)	109.5
H(28A)-C(28)-H(28C)	109.5
H(28B)-C(28)-H(28C)	109.5
C(30)-C(29)-Ru(1)	118.6(3)
C(30)-C(29)-H(29)	120.7
Ru(1)-C(29)-H(29)	120.7
C(31)-C(30)-C(35)	118.9(4)
C(31)-C(30)-C(29)	122.4(4)

C(35)-C(30)-C(29)	118.7(4)
C(32)-C(31)-C(30)	119.5(5)
C(32)-C(31)-H(31)	120.3
C(30)-C(31)-H(31)	120.3
C(33)-C(32)-C(31)	119.9(5)
C(33)-C(32)-H(32)	120.0
C(31)-C(32)-H(32)	120.0
C(32)-C(33)-C(34)	122.0(5)
C(32)-C(33)-H(33)	119.0
C(34)-C(33)-H(33)	119.0
C(33)-C(34)-C(35)	117.9(5)
C(33)-C(34)-H(34)	121.0
C(35)-C(34)-H(34)	121.0
O(1)-C(35)-C(34)	125.5(4)
O(1)-C(35)-C(30)	112.7(4)
C(34)-C(35)-C(30)	121.8(4)
O(2)-S(1)-O(3)	117.1(4)
O(2)-S(1)-O(4)	113.3(4)
O(3)-S(1)-O(4)	113.2(3)
O(2)-S(1)-C(36)	104.1(3)
O(3)-S(1)-C(36)	103.8(4)
O(4)-S(1)-C(36)	103.4(4)
F(2)-C(36)-F(1)	110.6(6)
F(2)-C(36)-F(3)	102.2(6)
F(1)-C(36)-F(3)	107.1(7)
F(2)-C(36)-S(1)	114.4(6)
F(1)-C(36)-S(1)	112.2(4)
F(3)-C(36)-S(1)	109.7(5)
O(7)-S(2)-O(6)	116.8(5)
O(7)-S(2)-O(5)	114.1(4)
O(6)-S(2)-O(5)	115.1(4)
O(7)-S(2)-C(37)	109.2(4)
O(6)-S(2)-C(37)	96.4(4)

O(5)-S(2)-C(37)	102.4(3)
F(6)-C(37)-F(4)	102.0(7)
F(6)-C(37)-F(5)	109.2(6)
F(4)-C(37)-F(5)	111.6(6)
F(6)-C(37)-S(2)	113.9(6)
F(4)-C(37)-S(2)	110.8(5)
F(5)-C(37)-S(2)	109.4(5)
O(7A)-S(2A)-O(5A)	125(3)
O(7A)-S(2A)-O(6A)	99.5(19)
O(5A)-S(2A)-O(6A)	118(2)
O(7A)-S(2A)-C(37A)	106.9(17)
O(5A)-S(2A)-C(37A)	99(2)
O(6A)-S(2A)-C(37A)	106.6(11)
F(4A)-C(37A)-F(5A)	110.0(12)
F(4A)-C(37A)-F(6A)	108.4(16)
F(5A)-C(37A)-F(6A)	108.0(15)
F(4A)-C(37A)-S(2A)	116(2)
F(5A)-C(37A)-S(2A)	111.8(18)
F(6A)-C(37A)-S(2A)	102(3)
C(1Z)-N(1Z)-C(2Z)	119.0(6)
C(1Z)-N(1Z)-C(3Z)	123.9(7)
C(2Z)-N(1Z)-C(3Z)	117.1(6)
O(1Z)-C(1Z)-N(1Z)	123.3(7)
O(1Z)-C(1Z)-H(1Z)	118.4
N(1Z)-C(1Z)-H(1Z)	118.4
N(1Z)-C(2Z)-H(2Z1)	109.5
N(1Z)-C(2Z)-H(2Z2)	109.5
H(2Z1)-C(2Z)-H(2Z2)	109.5
N(1Z)-C(2Z)-H(2Z3)	109.5
H(2Z1)-C(2Z)-H(2Z3)	109.5
H(2Z2)-C(2Z)-H(2Z3)	109.5
N(1Z)-C(3Z)-H(3Z1)	109.5
N(1Z)-C(3Z)-H(3Z2)	109.5

H(3Z1)-C(3Z)-H(3Z2)	109.5
N(1Z)-C(3Z)-H(3Z3)	109.5
H(3Z1)-C(3Z)-H(3Z3)	109.5
H(3Z2)-C(3Z)-H(3Z3)	109.5

Table S5: Anisotropic displacement parameters ($A^2 \times 10^3$). The anisotropic displacement factor exponent takes the form: $-2 \pi^2 [h^2 a^{*2} U_{11} + 2 h k a^* b^* U_{12}]$.

	U11	U22	U33	U23	U13	U12
Ru(1)	18(1)	13(1)	18(1)	4(1)	12(1)	4(1)
Cl(1)	48(1)	35(1)	32(1)	22(1)	25(1)	26(1)
Cl(2)	20(1)	35(1)	21(1)	1(1)	6(1)	10(1)
O(1)	29(2)	14(1)	33(2)	1(1)	19(2)	4(1)
C(1)	17(2)	20(2)	14(2)	7(2)	11(2)	8(2)
N(1)	25(2)	12(2)	24(2)	1(1)	17(2)	2(1)
N(2)	20(2)	15(2)	20(2)	3(1)	14(2)	6(1)
C(2)	30(2)	15(2)	26(2)	2(2)	18(2)	7(2)
N(3)	33(2)	28(2)	39(2)	5(2)	27(2)	-2(2)
C(3)	30(2)	18(2)	26(2)	0(2)	19(2)	5(2)
N(4)	18(2)	25(2)	26(2)	8(2)	12(2)	2(2)
C(4)	26(2)	10(2)	28(2)	3(2)	19(2)	1(2)
C(5)	25(2)	16(2)	28(2)	7(2)	17(2)	2(2)
C(6)	25(2)	23(2)	32(3)	7(2)	17(2)	3(2)
C(7)	30(3)	21(2)	30(3)	0(2)	22(2)	-4(2)
C(8)	33(3)	19(2)	23(2)	4(2)	17(2)	-5(2)
C(9)	30(2)	16(2)	23(2)	3(2)	14(2)	0(2)
C(10)	32(3)	38(3)	38(3)	18(2)	22(2)	10(2)
C(11)	37(3)	28(2)	26(3)	9(2)	12(2)	0(2)
C(12)	57(4)	41(3)	64(4)	-1(3)	47(4)	0(3)
C(13)	98(6)	35(3)	150(8)	25(4)	106(6)	8(4)
C(14)	66(3)	81(3)	72(3)	27(2)	39(2)	14(2)
C(15)	22(2)	19(2)	20(2)	6(2)	16(2)	8(2)
C(16)	16(2)	24(2)	19(2)	5(2)	12(2)	8(2)
C(17)	20(2)	24(2)	19(2)	9(2)	13(2)	9(2)
C(18)	16(2)	24(2)	21(2)	6(2)	13(2)	4(2)
C(19)	12(2)	34(2)	22(2)	8(2)	9(2)	7(2)

C(20)	21(2)	31(2)	19(2)	10(2)	14(2)	12(2)
C(21)	18(2)	29(2)	22(2)	10(2)	7(2)	5(2)
C(22)	24(2)	39(3)	29(3)	20(2)	15(2)	16(2)
C(23)	40(3)	23(2)	51(3)	14(2)	28(3)	6(2)
C(24)	16(2)	37(3)	32(3)	10(2)	6(2)	-2(2)
C(25)	24(2)	47(3)	27(3)	14(2)	15(2)	-3(2)
C(26)	82(4)	38(3)	102(5)	4(3)	68(4)	-5(3)
C(27)	189(12)	60(5)	131(9)	45(6)	101(9)	47(7)
C(28)	73(5)	26(3)	113(6)	9(3)	73(5)	-5(3)
C(29)	20(2)	19(2)	21(2)	5(2)	11(2)	8(2)
C(30)	31(2)	26(2)	26(2)	8(2)	19(2)	14(2)
C(31)	53(3)	38(3)	41(3)	12(2)	36(3)	19(3)
C(32)	79(5)	60(4)	52(4)	20(3)	53(4)	34(4)
C(33)	86(5)	41(3)	43(4)	1(3)	42(4)	28(3)
C(34)	53(3)	24(2)	32(3)	4(2)	21(3)	15(2)
C(35)	29(2)	27(2)	21(2)	7(2)	13(2)	14(2)
S(1)	31(1)	29(1)	73(1)	15(1)	30(1)	7(1)
O(2)	24(2)	119(5)	87(4)	-32(4)	23(3)	-5(3)
O(3)	74(3)	72(3)	75(4)	41(3)	27(3)	36(3)
O(4)	86(4)	78(4)	90(4)	47(3)	60(3)	15(3)
C(36)	41(4)	36(3)	102(6)	-17(4)	41(4)	-8(3)
F(1)	62(2)	37(2)	105(4)	-11(2)	42(3)	6(2)
F(2)	67(3)	134(4)	96(4)	-10(3)	70(3)	-13(3)
F(3)	46(2)	53(3)	184(6)	3(3)	22(3)	22(2)
S(2)	40(1)	33(1)	37(1)	8(1)	24(1)	4(1)
O(5)	38(3)	53(3)	49(3)	6(2)	30(3)	-4(2)
O(6)	89(5)	78(4)	41(3)	36(3)	12(3)	11(3)
O(7)	61(4)	45(3)	86(4)	15(3)	19(3)	29(3)
C(37)	51(3)	65(4)	66(4)	21(3)	31(3)	7(3)
F(4)	77(3)	66(3)	111(4)	10(2)	52(3)	25(2)
F(5)	35(2)	72(3)	75(3)	10(2)	19(2)	-1(2)
F(6)	113(4)	140(4)	83(3)	31(3)	69(3)	1(3)
S(2A)	31(4)	51(5)	41(4)	36(4)	23(3)	20(3)

O(5A)	87(17)	92(17)	88(17)	35(8)	42(9)	21(7)
O(6A)	40(6)	37(6)	19(6)	18(5)	17(5)	26(5)
O(7A)	70(13)	59(12)	69(13)	27(7)	23(7)	26(7)
C(37A)	51(3)	65(4)	66(4)	21(3)	31(3)	7(3)
F(4A)	77(3)	66(3)	111(4)	10(2)	52(3)	25(2)
F(5A)	76(9)	84(10)	79(10)	23(6)	37(7)	25(6)
F(6A)	113(4)	140(4)	83(3)	31(3)	69(3)	1(3)
N(1Z)	44(3)	41(3)	54(3)	15(2)	16(3)	14(2)
O(1Z)	42(3)	122(5)	90(4)	68(4)	34(3)	26(3)
C(1Z)	52(4)	95(6)	40(4)	15(4)	21(3)	42(4)
C(2Z)	58(4)	67(4)	48(4)	14(3)	25(3)	26(4)
C(3Z)	58(5)	58(5)	122(8)	34(5)	10(5)	13(4)

Table S6: Hydrogen coordinates (x 10^4) and isotropic displacement parameters (A^2 x 10^3).

	x	y	z	U(eq)
H(2A)	5357	4631	3912	28
H(2B)	6270	4533	4765	28
H(3A)	4989	3724	5052	30
H(3B)	4090	3944	4270	30
H(6)	9141	3546	4145	31
H(8)	7247	4047	1974	32
H(10A)	7530	3522	5145	49
H(10B)	8464	3044	5052	49
H(10C)	7229	2327	4532	49
H(11A)	5201	4323	2416	49
H(11B)	4806	3088	1895	49
H(11C)	5394	3847	1562	49
H(12A)	8881	2603	1956	82
H(12B)	9640	3465	1823	82
H(12C)	8378	3356	1486	82
H(13A)	10036	5309	2661	122
H(13B)	9578	5650	3373	122
H(13C)	8769	5167	2334	122
H(14A)	10150	3320	3524	108
H(14B)	10505	4548	4062	108
H(14C)	10839	4114	3283	108
H(17)	3472	147	4885	23
H(19)	1009	603	3022	28
H(21A)	5622	1922	4980	36
H(21B)	5315	1105	5397	36
H(21C)	5206	2252	5722	36
H(22A)	1325	1830	2373	40
H(22B)	2527	2381	2556	40

H(22C)	2061	2868	3230	40
H(23A)	1461	-1781	3014	53
H(23B)	1098	-2181	3663	53
H(23C)	2339	-1570	3997	53
H(24A)	73	-53	3626	48
H(24B)	-251	-1261	3486	48
H(24C)	68	-907	2797	48
H(25A)	2263	-363	5284	50
H(25B)	998	-913	4920	50
H(25C)	1431	307	5136	50
H(26)	2981	-2172	885	88
H(27A)	4847	-2499	1741	169
H(27B)	4079	-2874	2153	169
H(27C)	4967	-1752	2671	169
H(28A)	3539	-1141	2736	100
H(28B)	2460	-2071	2030	100
H(28C)	2603	-934	1984	100
H(29)	6038	1736	2257	24
H(31)	6676	897	1123	47
H(32)	6540	-594	20	65
H(33)	5307	-2170	-368	65
H(34)	4215	-2327	345	44
H(1Z)	8503	-514	1468	74
H(2Z1)	8861	1164	3660	87
H(2Z2)	7567	820	3237	87
H(2Z3)	8182	1515	2877	87
H(3Z1)	7565	-1553	1948	134
H(3Z2)	6806	-1000	2285	134
H(3Z3)	7909	-1058	2989	134

Table S7: Torsion angles [°].

C(29)-Ru(1)-O(1)-C(35)	-0.7(3)
C(1)-Ru(1)-O(1)-C(35)	115(4)
Cl(2)-Ru(1)-O(1)-C(35)	99.4(3)
Cl(1)-Ru(1)-O(1)-C(35)	-100.0(3)
C(29)-Ru(1)-O(1)-C(26)	173.7(6)
C(1)-Ru(1)-O(1)-C(26)	-70(4)
Cl(2)-Ru(1)-O(1)-C(26)	-86.1(6)
Cl(1)-Ru(1)-O(1)-C(26)	74.5(6)
C(29)-Ru(1)-C(1)-N(1)	-4.0(5)
O(1)-Ru(1)-C(1)-N(1)	-120(4)
Cl(2)-Ru(1)-C(1)-N(1)	-103.8(4)
Cl(1)-Ru(1)-C(1)-N(1)	95.7(4)
C(29)-Ru(1)-C(1)-N(2)	172.1(3)
O(1)-Ru(1)-C(1)-N(2)	56(4)
Cl(2)-Ru(1)-C(1)-N(2)	72.3(3)
Cl(1)-Ru(1)-C(1)-N(2)	-88.2(3)
N(2)-C(1)-N(1)-C(4)	173.8(4)
Ru(1)-C(1)-N(1)-C(4)	-9.7(7)
N(2)-C(1)-N(1)-C(2)	-1.8(5)
Ru(1)-C(1)-N(1)-C(2)	174.7(3)
N(1)-C(1)-N(2)-C(15)	-170.7(4)
Ru(1)-C(1)-N(2)-C(15)	12.2(5)
N(1)-C(1)-N(2)-C(3)	-2.9(5)
Ru(1)-C(1)-N(2)-C(3)	-180.0(3)
C(1)-N(1)-C(2)-C(3)	5.3(5)
C(4)-N(1)-C(2)-C(3)	-170.8(4)
C(1)-N(2)-C(3)-C(2)	6.0(5)
C(15)-N(2)-C(3)-C(2)	174.2(4)
N(1)-C(2)-C(3)-N(2)	-6.2(4)
C(1)-N(1)-C(4)-C(5)	-87.1(5)
C(2)-N(1)-C(4)-C(5)	88.3(5)

C(1)-N(1)-C(4)-C(9)	96.2(5)
C(2)-N(1)-C(4)-C(9)	-88.3(5)
C(9)-C(4)-C(5)-C(6)	0.4(6)
N(1)-C(4)-C(5)-C(6)	-176.1(4)
C(9)-C(4)-C(5)-C(10)	-178.9(4)
N(1)-C(4)-C(5)-C(10)	4.5(6)
C(4)-C(5)-C(6)-C(7)	-1.3(6)
C(10)-C(5)-C(6)-C(7)	178.0(4)
C(5)-C(6)-C(7)-C(8)	0.9(7)
C(5)-C(6)-C(7)-N(3)	179.7(4)
C(12)-N(3)-C(7)-C(8)	-62.6(6)
C(14)-N(3)-C(7)-C(8)	178.8(5)
C(13)-N(3)-C(7)-C(8)	56.7(6)
C(12)-N(3)-C(7)-C(6)	118.5(5)
C(14)-N(3)-C(7)-C(6)	-0.1(7)
C(13)-N(3)-C(7)-C(6)	-122.2(6)
C(6)-C(7)-C(8)-C(9)	0.5(7)
N(3)-C(7)-C(8)-C(9)	-178.4(4)
C(7)-C(8)-C(9)-C(4)	-1.4(6)
C(7)-C(8)-C(9)-C(11)	179.4(4)
C(5)-C(4)-C(9)-C(8)	0.9(6)
N(1)-C(4)-C(9)-C(8)	177.5(4)
C(5)-C(4)-C(9)-C(11)	-179.8(4)
N(1)-C(4)-C(9)-C(11)	-3.2(6)
C(1)-N(2)-C(15)-C(16)	80.9(5)
C(3)-N(2)-C(15)-C(16)	-86.1(5)
C(1)-N(2)-C(15)-C(20)	-105.5(5)
C(3)-N(2)-C(15)-C(20)	87.4(5)
C(20)-C(15)-C(16)-C(17)	9.5(6)
N(2)-C(15)-C(16)-C(17)	-177.2(3)
C(20)-C(15)-C(16)-C(21)	-164.5(4)
N(2)-C(15)-C(16)-C(21)	8.8(6)
C(15)-C(16)-C(17)-C(18)	-3.9(6)

C(21)-C(16)-C(17)-C(18)	170.4(4)
C(16)-C(17)-C(18)-C(19)	-1.9(6)
C(16)-C(17)-C(18)-N(4)	-179.9(4)
C(24)-N(4)-C(18)-C(19)	-5.5(6)
C(23)-N(4)-C(18)-C(19)	115.0(5)
C(25)-N(4)-C(18)-C(19)	-124.0(4)
C(24)-N(4)-C(18)-C(17)	172.5(4)
C(23)-N(4)-C(18)-C(17)	-67.0(5)
C(25)-N(4)-C(18)-C(17)	54.0(5)
C(17)-C(18)-C(19)-C(20)	2.4(6)
N(4)-C(18)-C(19)-C(20)	-179.7(4)
C(16)-C(15)-C(20)-C(19)	-9.0(6)
N(2)-C(15)-C(20)-C(19)	177.6(4)
C(16)-C(15)-C(20)-C(22)	167.1(4)
N(2)-C(15)-C(20)-C(22)	-6.3(6)
C(18)-C(19)-C(20)-C(15)	3.0(6)
C(18)-C(19)-C(20)-C(22)	-173.3(4)
C(35)-O(1)-C(26)-C(28)	178.7(6)
Ru(1)-O(1)-C(26)-C(28)	4.6(10)
C(35)-O(1)-C(26)-C(27)	72.8(7)
Ru(1)-O(1)-C(26)-C(27)	-101.3(5)
C(1)-Ru(1)-C(29)-C(30)	-178.0(3)
O(1)-Ru(1)-C(29)-C(30)	0.2(3)
Cl(2)-Ru(1)-C(29)-C(30)	-84.6(3)
Cl(1)-Ru(1)-C(29)-C(30)	84.3(3)
Ru(1)-C(29)-C(30)-C(31)	178.7(4)
Ru(1)-C(29)-C(30)-C(35)	0.4(6)
C(35)-C(30)-C(31)-C(32)	-0.2(8)
C(29)-C(30)-C(31)-C(32)	-178.5(5)
C(30)-C(31)-C(32)-C(33)	0.7(10)
C(31)-C(32)-C(33)-C(34)	-0.9(11)
C(32)-C(33)-C(34)-C(35)	0.5(10)
C(26)-O(1)-C(35)-C(34)	6.7(8)

Ru(1)-O(1)-C(35)-C(34)	-178.1(4)
C(26)-O(1)-C(35)-C(30)	-174.1(6)
Ru(1)-O(1)-C(35)-C(30)	1.1(5)
C(33)-C(34)-C(35)-O(1)	179.1(5)
C(33)-C(34)-C(35)-C(30)	0.0(8)
C(31)-C(30)-C(35)-O(1)	-179.4(4)
C(29)-C(30)-C(35)-O(1)	-1.0(6)
C(31)-C(30)-C(35)-C(34)	-0.1(8)
C(29)-C(30)-C(35)-C(34)	178.2(5)
O(2)-S(1)-C(36)-F(2)	-58.1(7)
O(3)-S(1)-C(36)-F(2)	64.9(6)
O(4)-S(1)-C(36)-F(2)	-176.8(5)
O(2)-S(1)-C(36)-F(1)	174.8(7)
O(3)-S(1)-C(36)-F(1)	-62.2(7)
O(4)-S(1)-C(36)-F(1)	56.1(7)
O(2)-S(1)-C(36)-F(3)	55.9(7)
O(3)-S(1)-C(36)-F(3)	178.9(5)
O(4)-S(1)-C(36)-F(3)	-62.8(6)
O(7)-S(2)-C(37)-F(6)	-64.0(7)
O(6)-S(2)-C(37)-F(6)	174.8(6)
O(5)-S(2)-C(37)-F(6)	57.3(7)
O(7)-S(2)-C(37)-F(4)	-178.2(6)
O(6)-S(2)-C(37)-F(4)	60.5(6)
O(5)-S(2)-C(37)-F(4)	-57.0(7)
O(7)-S(2)-C(37)-F(5)	58.4(7)
O(6)-S(2)-C(37)-F(5)	-62.8(6)
O(5)-S(2)-C(37)-F(5)	179.7(5)
O(7A)-S(2A)-C(37A)-F(4A)	-176(2)
O(5A)-S(2A)-C(37A)-F(4A)	52(3)
O(6A)-S(2A)-C(37A)-F(4A)	-71(2)
O(7A)-S(2A)-C(37A)-F(5A)	-49(3)
O(5A)-S(2A)-C(37A)-F(5A)	179(3)
O(6A)-S(2A)-C(37A)-F(5A)	56(2)

O(7A)-S(2A)-C(37A)-F(6A)	66(2)
O(5A)-S(2A)-C(37A)-F(6A)	-65(3)
O(6A)-S(2A)-C(37A)-F(6A)	171.8(17)
C(2Z)-N(1Z)-C(1Z)-O(1Z)	4.4(10)
C(3Z)-N(1Z)-C(1Z)-O(1Z)	-177.7(7)
